# Microenvironmental networks promote tumor heterogeneity and enrich for metastatic cancer stem-like cells in Luminal-A breast tumor cells

**DOI:** 10.18632/oncotarget.13213

**Published:** 2016-11-08

**Authors:** Polina Weitzenfeld, Tsipi Meshel, Adit Ben-Baruch

**Affiliations:** ^1^ Department of Cell Research and Immunology, George S. Wise Faculty of Life Sciences, Tel Aviv University, Tel Aviv, Israel

**Keywords:** adhesion molecules, luminal-A breast cancer, cancer stem cells, metastasis, tumor microenvironment

## Abstract

The roles of the tumor microenvironment (TME) in generating intra-tumoral diversity within each specific breast cancer subtype are far from being fully elucidated. In this study, we exposed Luminal-A breast cancer cells in culture to combined “TME Stimulation”, representing three typical arms of the breast TME: hormonal (estrogen), inflammatory (tumor necrosis factor α) and growth-promoting (epidermal growth factor). In addition to enriching the tumor cell population with CD44+/β1+ cells (as we previously published), TME Stimulation selected for CD44+/CD24^low/−^ stem-like cells, that were further enriched by doxorubicin treatment and demonstrated high plasticity *in vitro* and *in vivo*. Knock-down experiments revealed that CD44 and Zeb1 regulated CD24 and β1 expression and controlled differently cell spreading and formation of cellular protrusions. TME-enriched CD44+/CD24^low/−^ stem-like cells promoted dissemination to bones and lymph nodes, whereas CD44+/β1+ cells had a low metastatic potential. Mixed co-injections of TME-enriched CD44+/CD24^low/−^ and CD44+/β1+ sub-populations generated metastases populated mostly by CD44+/CD24^low/−^-derived cells. Thus, combined activities of several TME factors select for CD44+/CD24^low/−^ stem-like cells that dictate the metastatic phenotype of Luminal-A breast tumor cells, suggesting that therapeutic modalities targeting the TME could be introduced as a potential strategy of inhibiting the detrimental stem-like sub-population in this disease subtype.

## INTRODUCTION

Many solid tumors are characterized by heterogeneity that impinges on tumor progression, prognosis and therapy. In breast cancer, the inter-tumoral heterogeneity - manifested by distinct histological, molecular and genetic characteristics of tumors - has strong clinical implications, leading to categorization of disease into several subtypes, including Luminal-A, Luminal-B, HER2+ and Triple Negative (TN, often interchanged with “Basal”) [1–4].

However, not only inter-tumoral heterogeneity is typical of breast tumors but also intra-tumoral heterogeneity that can dictate the metastatic potential of the cells and their resistance to therapy [5]. Such intra-tumoral heterogeneity is reflected by varying expression levels of defined markers within the same tumor [such as estrogen receptors (ER)] and also by clonal genetic diversity within tumors [5–7].

Recent studies connect intra-tumoral heterogeneity with the unique subset of cancer stem cells (CSCs), often referred to as cancer initiating cells. The term CSCs reflects the potential of these cells to self-renew and reconstitute the entire tumor mass, with its phenotypic heterogeneity, when transplanted to mice [8, 9]. Increasing evidence indicates that the CSC sub-population is the one that initiates metastases and provides resistance to chemotherapy [10, 11].

The origins of CSCs are currently under extensive investigation and two main models have been proposed so far [12–14]: The “hierarchical model” suggests that CSCs arise from normal stem cells that underwent malignant transformation, thus they cannot originate from differentiated tumor cells. The alternative “plastic model” states that any of the tumor cells may de-differentiate and revert to a stem-like state; thus, the pool of CSCs is continuously regenerated from the plastic non-CSC pool. The fact that cancer cells undergoing epithelial-to-mesenchymal transition (EMT) also express markers associated with CSCs supports this latter model [15–18].

Genetic alterations have been identified as strong drivers of tumor cell heterogeneity and regulation of CSCs. In parallel, signaling cascades that are activated by exogenous ligands/pathways, such as Wnt/β-catenin, Notch, Hedgehog and transforming growth factor β have been shown to contribute to tumor heterogeneity and to generation of CSCs [19, 20]. While these findings suggest key roles for the tumor microenvironment (TME) in regulating intra-tumoral plasticity, many aspects of this process are yet to be revealed. Specifically, it is important to determine how cancer cells of specific disease subtype/s develop into heterogeneous tumor sub-populations and what are the roles of the TME in generating CSCs in each subtype of disease.

To address these aspects, the aim of our study was to determine how intra-tumoral heterogeneity in Luminal-A breast tumor cells is affected by factors of the TME. The response of the tumor cells would depend on their ability to interpret the TME signals by the appropriate receptors (which may be typical of this subtype, such as ERs in luminal tumors) and on the content of specific factors at the TME. In our study, we chose to expose the tumor cells to simultaneous stimulation by representatives of three arms of the TME: (1) The hormonal arm–represented by estrogen, a key driver of tumor cell proliferation and survival in luminal tumors [1, 21]; (2) The inflammatory arm–represented by the inflammatory cytokine tumor necrosis factor α (TNFα). Chronic expression of TNFα at the TME was highly linked to cancer progression. TNFα has direct tumor-promoting effects and is expressed by ~90% of recurrent breast cancers, including of the Luminal-A subtype. Accordingly, inhibition of TFNα leads to reduced tumor growth and metastasis in animal models of breast cancer, including of the Luminal-A subtype [22–32]; (3) The growth-stimulating arm–Epidermal growth factor (EGF) in expressed in breast carcinomas, particularly in tumors of the luminal subtype [33–37]. In parallel, luminal breast tumor cells express, at various levels, members of the EGF receptor family (EGFRs), including EGFR and HER2. Cross-talk between the EGF and the estrogen pathways can potentiate genomic and non-genomic signaling of the ER, and activation of the EGF pathway contributes to the development of endocrine resistance in Luminal-A patients [38, 39].

In our published studies we demonstrated that the exposure of Luminal-A breast tumor cells to combined “TME Stimulation” by estrogen+TNFα+EGF for three days in culture had driven a high *in vivo* metastatic phenotype in Luminal-A breast tumor cells [40, 41]. Moreover, following such stimulation the metastatic pattern of the tumor cells was re-shaped and shifted from lymphatic dissemination towards the more aggressive bone-metastasizing phenotype [41]. *In vitro*, the joint activity of the three factors, applied together as TME Stimulation (estrogen+TNFα+EGF), was much more potent than of each factor alone, leading to tumor cell remodeling towards an EMT-like phenotype and increased tumor cell scattering. In parallel, a unique CD44+/β1+ sub-population of tumor cells has been enriched by TME Stimulation, identified by the simultaneous expression of two adhesion molecules, CD44 and the β1 integrin [40].

Elevated expression of β1 [42, 43], CD44 [44–46] or both combined [47] by the cancer cells may promote tumor cell adhesion and lead to increased tumor aggressiveness. However, high CD44 expression levels also characterize the CSC sub-population in breast cancer, agreed by most researchers as having the CD44+/CD24^low/−^ phenotype. Thus, it is possible that when Luminal-A breast tumor cells are exposed to multiple factors of the TME simultaneously - as recapitulated in our study by the combined TME Stimulation - the CSC sub-population is enriched in parallel to the CD44+/β1+ sub-population.

Indeed, in this study we demonstrate that the combined TME Stimulation by estrogen+TNFα+EGF has enriched Luminal-A breast tumor cells not only for the CD44+/β1+ sub-population, but also for the CD44+/CD24^low/−^ sub-population. By that, factors of the TME demonstrate high ability to promote intra-tumoral heterogeneity. We identified partial overlap between the TME-enriched CD44+/β1+ and CD44+/CD24^low/−^ sub-populations and further characterized the CD44+/CD24^low/−^ sub-population as having a stem-like phenotype. Also, we identified several molecular pathways that regulate the propagation and maintenance of these two distinct sub-populations. *In vivo*, our findings indicated that although both sub-populations had similar abilities to form primary tumors, the stem-like sub-population of CD44+/CD24^low/−^ cells was the one that had led to metastatic dissemination, while cells with the CD44+/β1+ phenotype were hardly metastatic.

Overall, our findings provide evidence to key roles of the TME in generating intra-tumoral heterogeneity and in enriching the stem-like cell sub-population in the Luminal-A subtype of breast cancer. Although several tumor cell sub-populations may emerge out of TME stimulation, it is the stem-like subset that dictates the metastatic phenotype of Luminal-A breast cancer cells, positioning the TME as an attractive target in the design of improved therapeutics for Luminal-A breast cancer.

## RESULTS

### Exposure of Luminal-A breast tumor cells to TME Stimulation enriches not only for the CD44+/β1+ sub-population but also for CD44+/CD24^low/−^ cells

To determine the impact of combined TME Stimulation (estrogen+TNFα+EGF) on tumor cell heterogeneity, two very well-established human Luminal-A breast tumor cell lines were used in our current study, MCF-7 and T47D cells. MCF-7 and T47D cells were found to adhere well to the needs of this study, as they are able to respond to TME Stimulation, composed of estrogen+TNFα+EGF [40, 41], by: (1) Expressing ER and responding to estrogen; (2) Expressing TNFα receptors and responding to TNFα; (3) Responding to EGF, through the expression of different EGF receptors [40, 48–51].

**Figure 1:**
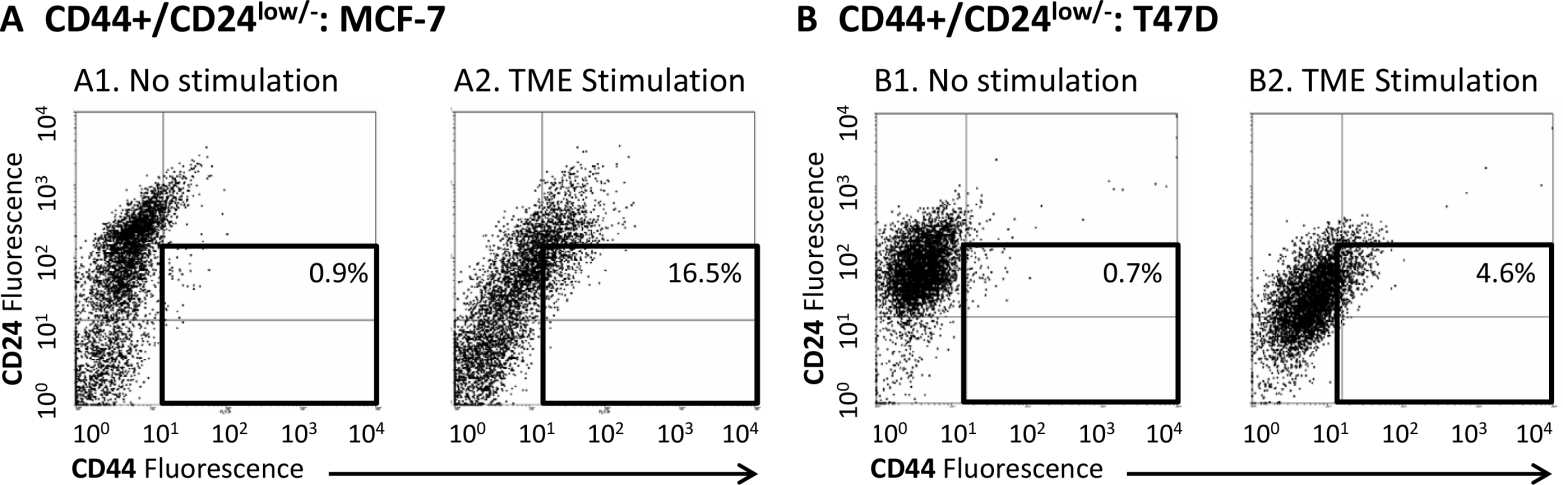
TME Stimulation enriches the CD44+/CD24^low/−^ sub-population in MCF-7 and T47D Luminal-A breast tumor cells MCF-7 or T47D breast tumor cells were exposed to TME Stimulation in culture (estrogen - 10^−8^ M, TNFα - 50 ng/ml, EGF - 30 ng/ml) for three days. No stimulation = Cells grown with vehicles only. Expression of CD44 and CD24 on the surface of the cells was determined by FACS analyses, using fluorescently-labeled specific Abs. Isotype-matched control Abs were used in order to determine baseline staining and to set location of axes (Data not shown; Please see “Materials and methods” for more details). (**A**) MCF-7 cells. (**B**) T47D cells. (A1, B1) Non-stimulated cells. (A2, B2) Cells exposed to TME Stimulation. In both panels, the results are from a representative experiment of *n* ≥ 3, showing similar results. Averages ± SD obtained in *n* ≥ 3 independent experimental repeats are demonstrated in Supplementary Figure S2A.

In our previously published studies [40, 41] we demonstrated that combined TME Stimulation (for three days in culture) has increased the proportion of cells with the CD44+/β1+ phenotype in Luminal-A MCF-7 cells from 12.5 ± 3% to 44.3 ± 17.7% (range 23–63%) and in T47D cells from 5.1 ± 1.6 to 20.7 ± 5.7 (range 18–27%) (for the sake of clarity, the summary of these findings is presented in Supplementary Figure S1). The CD44 molecule is predominantly involved in adhesion, but also characterizes the CSC sub-population in human breast cancer, denoted by the CD44+/CD24^low/−^ phenotype. Therefore, we determined the impact of TME Stimulation on the proportion of CD44+/CD24^low/−^ cells and found that exposure to TME factors increased the proportion of the CD44+/CD24^low/−^ sub-population in MCF-7 cells from 0.9±0.5% (range 0.2–1.6%) to 13.8 ± 6.9% (range 7.1–25.6%) (Figure 1A; Supplementary Figure S2A1), and in T47D cells from 0.3 ± 0.3% (range 0.1–0.7%) to 5.5 ± 2.1% (range 4.0–7.9%) (Figure 1B; Supplementary Figure S2A2) (The impact of TME Stimulation on the expression of CD24 alone in MCF-7 an T47D cells is demonstrated in Supplementary Figure S2B).

Thus, in response to TME Stimulation, two cell sub-populations were enriched in MCF-7 and T47D Luminal-A breast tumor cells: cells with the CD44+/β1+ phenotype and cells with the CD44+/CD24^low/−^ phenotype, the latter possibly representing a sub-population of CSCs. Of note, following TME Stimulation the prevalence of CD44+/β1+ and of CD44+/CD24^low/−^ cells was higher in MCF-7 cells than in T47D cells (Supplementary Figure S1 and S2A). These results suggest that MCF-7 cells are more responsive than T47D cell to TME Stimulation, leading us to focus in the next parts of the study on MCF-7 cells only.

### The CD44+/β1+ and CD44+/CD24^low/−^ sub-populations partly overlap and exhibit high plasticity

**Figure 2:**
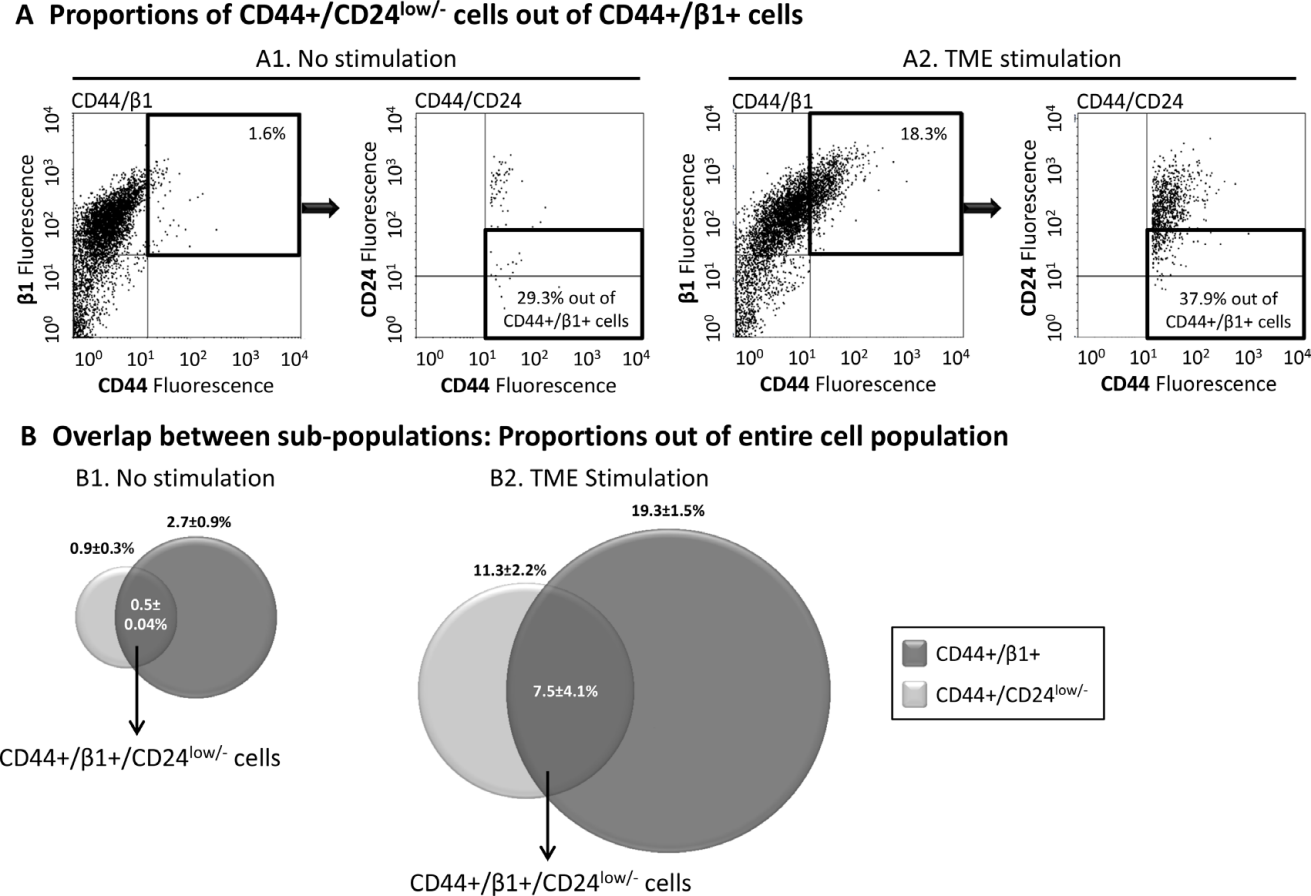
The TME-enriched CD44+/β1+ and CD44+/CD24^low/−^ sub-populations are partly overlapping MCF-7 breast tumor cells were exposed to TME Stimulation (as in Figure 1). No stimulation = Cells grown with vehicles only. Expression of CD44, β1 and/or CD24 was determined by FACS analyses, using fluorescently-labeled specific Abs. Isotype-matched Abs were used in order to determine baseline staining and to set location of axes (Data not shown; Please see “Materials and methods” for more details). (**A**) Dot plots demonstrating the proportion of CD44+/CD24^low/−^ cells out of CD44+/β1+ cells. (A1) Non-stimulated cells. (A2) Cells exposed to TME Stimulation (Please note: The percentages of CD44+/β1+ cells in these experiments were ~20%, which is at the lower end of the 23–63% range presented in Supplementary Figure S1A. The somewhat lower proportion of this sub-population in this figure may be due to some technical issues during the triple-dye fluorescence analysis). The results are from a representative experiment of *n* = 3, showing similar results, and their sum up is shown in Figure 2B. (**B**) Averages ± SD of overlapping and original sub-population frequencies out of the entire population of non-stimulated cells (B1) or of cells that were exposed to TME Stimulation (B2).

Being that CD44 is a common denominator of the above-described two sub-populations, we asked if there is an overlap between them and whether some of the cells that express high CD44 and high β1 levels (CD44+/β1+) are also CD24^low/−^, evidently giving rise to a unique CD44+/β1+/CD24^low/−^ sub-population. The results of Figure 2 indicate that there was some degree of overlap between the two sub-populations, particularly after TME Stimulation, because following such stimulation, 38.1 ± 18% of the CD44+/β1+ cells included the CD44+/CD24^low/−^ sub-population. As a result of this overlap, a certain percentage of cells demonstrated the CD44+/β1+/CD24^low/−^ phenotype: 0.5 ± 0.04% of the whole cell population of non-stimulated cells (0.5%, 0.6% and 0.5% in *n*= 3 independent experimental repeats) and 7.5 ± 4.1% of the whole cell population following TME Stimulation (6.9%, 3.7% and 11.8%, in the respective *n* = 3 experimental repeats) (Figure 2B).

In view of many reports about tumor cell plasticity, we asked whether the TME-enriched sub-populations maintain their distinct phenotypes over time, or do they drift back to their initial characteristics. In a series of experiments performed on TME-stimulated MCF-7 cells, we found that a phenotypic drift occurs in both sub-populations: CD44+/β1+ and CD44+/CD24^low/−^. Immediately after three days of TME Stimulation, 41 ± 17.4% and 18.7 ± 6.3% of tumor cells were characterized by the CD44+/β1+ and CD44+/CD24^low/−^ phenotypes, respectively (Table 1A). By separate processes of sorting, each of the two cell populations was enriched to ~100% and regrown in culture. FACS analyses, performed one week later, demonstrated that neither the CD44+/β1+ phenotype nor the CD44+/CD24^low/−^ phenotype were fully retained; only a small fraction of the tumor cells remained CD44+/β1+ or CD44+/CD24^low/−^ (2.0 ± 0.2% and 2.9 ± 1.6%, respectively; Table 1A), demonstrating that with time, the TME-enriched cell populations undergoes a phenotypic drift.

**Table 1:** A. Phenotypic drift: *In vitro*

Phenotype	Percentage: Three days after TME Stimulation	Percentage: After sorting	
		Immediately after sorting	After 1 week in culture
CD44+/β1+	41.0 ± 17.4%	100%	2.0 ± 0.2%
CD44+/CD24^low/−^	18.7 ± 6.3%	100%	2.9 ± 1.6%

B. Phenotypic drift: *In vivo*	
Phenotype of inoculated cells	Percentage: In dissociated tumors (out of tumor cells)
CD44+/β1+	9.0 ± 2.9%
CD44+/CD24^low/−^	4.5 ± 1.1%

MCF-7 breast tumor cells were exposed to TME Stimulation (as in Figure 1). Co-expression of CD44 and β1 or of CD44 and CD24 on the surface of the cells was determined by FACS analyses, using fluorescently-labeled specific Abs. Isotype-matched Abs were used as control (Data not shown). Proportions of the sub-populations characterized by the phenotype CD44+/β1+ or CD44+/CD24^low/−^ cells were determined. (A) Phenotypic drift *in vitro*. Following sorting and enrichment to ~100% of CD44+/β1+ or CD44+/CD24^low/−^, sorted cells were regrown in culture for one week. Then, the proportions of CD44+/β1+ or CD44+/CD24^low/−^ cells were determined by FACS analyses, respectively. The table sums up the results obtained in *n* ≥ 2 experimental repeats, showing similar results. (B) Phenotypic drift *in vivo*. Following sorting, each of the two sub-populations - CD44+/β1+ or CD44+/CD24^low/−^ - was inoculated orthotopically to the mammary fat pad of mice (the tumors were generated as part of the *in vivo* experiments described in Figures 8). At the endpoint of experiment, primary tumors were dissociated, and stained by fluorescently-labeled Abs to determine the proportion of CD44+/β1+ cells or CD44+/CD24^low/−^ cells, out of all tumor cells (gated by mPlum+ or mCherry+, as appropriate). The table sums up the results obtained in a total of *n* = 4–5 mice/group (tumor cells were administered to mice in 2 separate sets of injections).

To further explore this phenotypic drift, each of the two TME-enriched sub-populations was sorted to ~100% CD44+/β1+ cells in the first set of experiments or to ~100% CD44+/CD24^low/−^ cells in the second set, and were inoculated to the mammary fat pad of mice. At the endpoint of experiment, 12 weeks post inoculation, mice were euthanized, the primary tumors were resected, dissociated and the proportions of different cell types were determined. In tumors generated by cells that were purely of the mPlum-expressing CD44+/β1+ phenotype, mPlum-expressing tumor cells consisted 27.5 ± 3.2% of the tumor mass (the rest are expected to be various stromal cells and leukocytes). FACS analyses using the relevant antibodies demonstrated that out of these mPlum-expressing tumor cells, only 9.0 ± 2.9% retained the original phenotype of CD44+/β1+ (Table 1B). Similarly, following the inoculation of pure mCherry-expressing CD44+/CD24^low/−^ cells to mice, the tumors consisted of 52 ± 6.9% mCherry-expressing cells but only 4.5 ± 1.1% of the mCherry-expressing tumor cells retained the CD44+/CD24^low/−^ phenotype (Table 1B). These findings agree with the *in vitro* results, demonstrating high plasticity of the TME-enriched sub-populations and their ability to drift back, with time, to the relatively low numbers of each sub-population existing in the original cell cultures.

### TME Stimulation does not enrich for ALDH1+ cells but selects for chemotherapy-resistant CD44+/CD24^low/−^ cells

The CD44+/CD24^low/−^ phenotype is accepted by many researchers as a characteristic of the CSC sub-population in breast cancer. The ALDH1 enzyme is another stem cell marker which is particularly useful is studies of CSCs in culture, when contamination by other stem cells (such as hematopoietic stem cells) is not relevant [52, 53]. However, it was recently proposed that there are two distinct CSC sub-populations in breast cancer, one identified as CD44+/CD24^low/−^ and the other as ALDH1+ [54].

**Figure 3:**
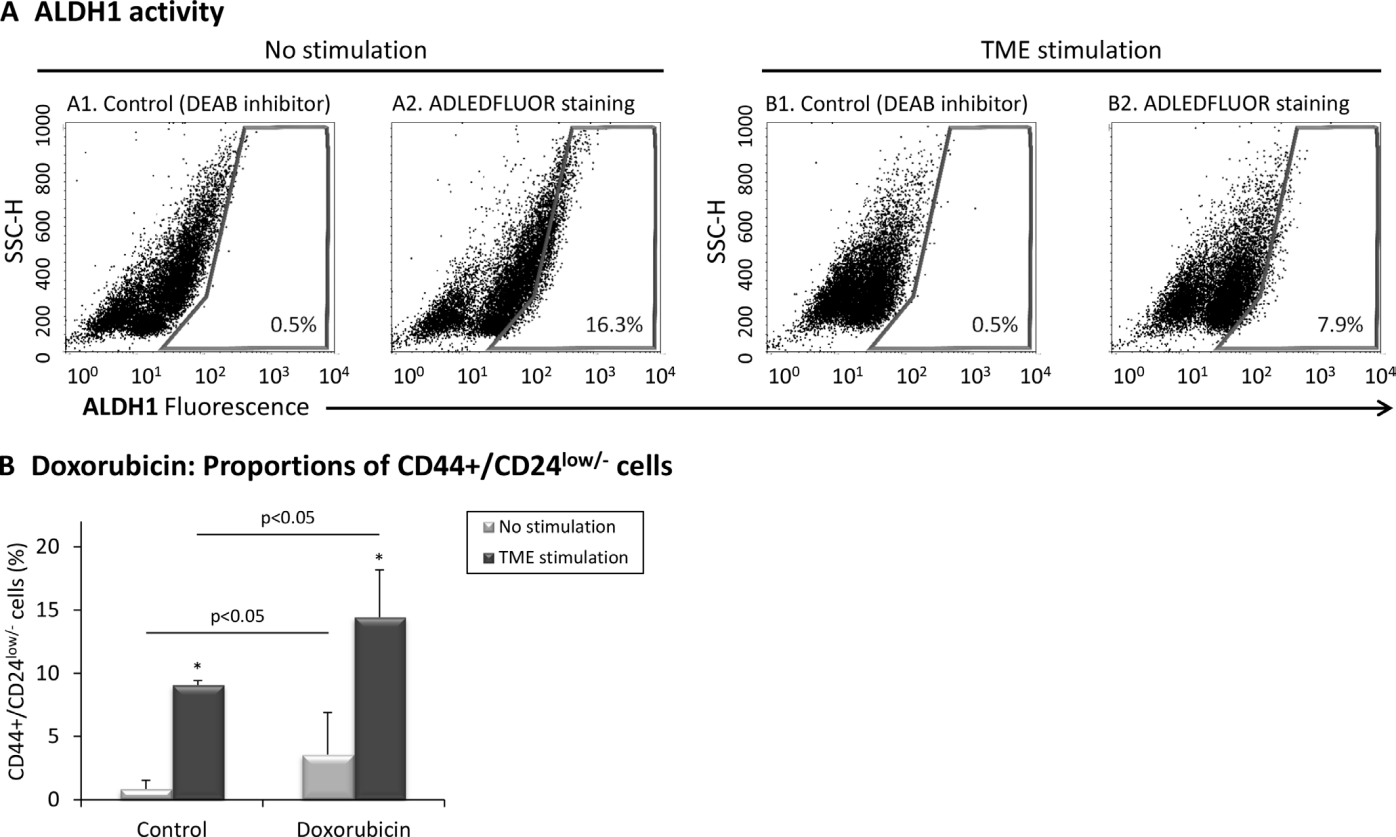
TME stimulation does not enrich for ALDH1+ cells but selects for doxorubicin-resistant CD44+/CD24^low/−^ cells (**A**) MCF-7 breast tumor cells were exposed to TME Stimulation (as in Figure 1). No stimulation = Cells grown with vehicles only. ALDH1 activity was determined using the ALDEFLUOR™ kit, where controls with DEAB inhibitor were used to set polygon gate location. (A1, A2) Non-stimulated cells. (B1, B2) Cells exposed to TME Stimulation. The results are from a representative experiment of *n* ≥ 3, showing similar results. Averages ± SD obtained in *n* ≥ 3 independent repeats are demonstrated in Supplementary Figure S3A and positive technical control of SKBR3 cells is shown in Supplementary Figure S3B. (**B**) Proportions of CD44+/CD24^low/−^ cells following treatment by doxorubicin. MCF-7 cells were concomitantly exposed to TME Stimulation (as in Figure 1) and to 0.1 μM doxorubicin (or vehicles as controls). The proportion of CD44+/CD24^low/−^ cells was determined by FACS analyses, using fluorescently-labeled specific Abs. Isotype-matched Abs were used as control (Data not shown). *p < 0.05 for the difference between TME-stimulated and non-stimulated cells. The panel sums up the results obtained in *n* = 3 independent repeats. Results of a representative experiment out of *n* = 3 experiments, showing similar findings, are presented in Supplementary Figure S4.

In line with these studies, we found that the proportion of ALDH1+ cells was not increased by TME Stimulation (Figure 3A). Of note, agreeing with published studies of MCF-7 cells [55, 56], our analyses indicated that the proportion of ALDH1+ cells in the original cell population was very low (See “No Stimulation” in Figure 3A and Supplementary Figure S3A; The SKBR3 technical positive control performed as expected, and stained nicely, as shown in Supplementary Figure S3B). Overall, the findings described above suggest that TME Stimulation enriches MCF-7 cells for a CD44+/CD24^low/−^ stem-like sub-population that is negative for ALDH1 expression, being in line with the phenotypes of CSCs in other studies [54].

To further characterize the CD44+/CD24^low/−^ sub-population, we followed previous studies demonstrating that chemotherapies select for CSCs [57]. Indeed, following treatment by doxorubicin (commonly used in the treatment of Luminal-A patients [58, 59]), the proportion of CD44+/CD24^low/−^ cells was much increased in TME-Stimulated cells compared to vehicle-treated control cells (Figure 3B and Supplementary Figure S4). These findings add to our published findings demonstrating that TME Stimulation increases resistance to chemotherapy [40] and suggest that TME-enriched stem-like CD44+/CD24^low/−^ cells contribute to reduced sensitivity to chemotherapeutic drugs.

The findings presented thus far suggest that CD44+/CD24^low/−^ cells may in fact be CSCs. These cells express surface markers that characterize CSCs in breast cancer and demonstrate other characteristics associated with the CSC phenotype: high plasticity *in vitro* and *in vivo*, and enrichment by cytotoxic agents. However, out of caution, we will term these cells “stem-like cells” throughout the following parts of the study.

### Identification of molecular mechanisms regulating the surface markers of CD44+/β1+ and CD44+/CD24^low/−^ sub-populations and of their impact on properties of cell spreading and generation of cellular protrusions

**Figure 4:**
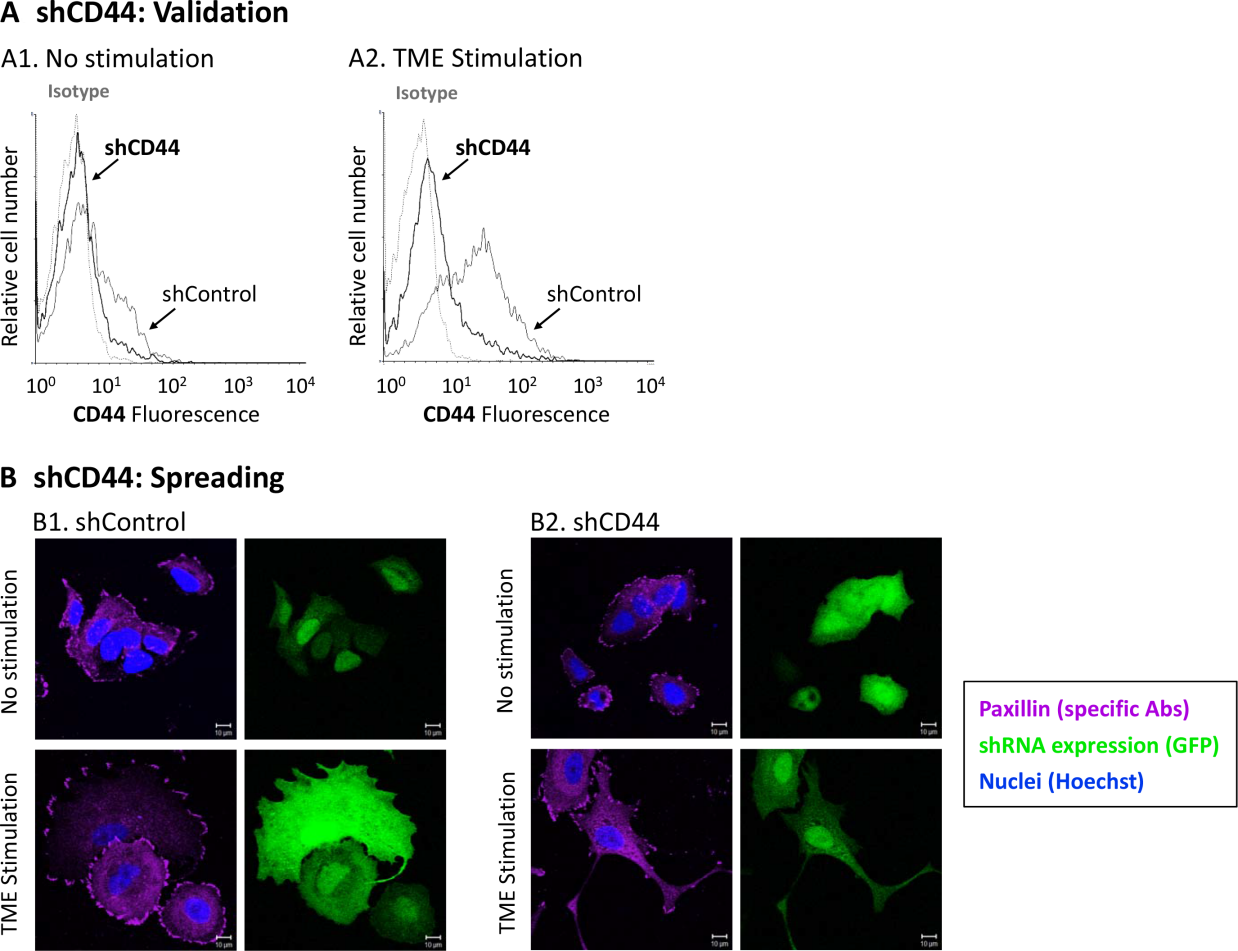
CD44 knock-down interferes with TME-induced cell spreading MCF-7 breast tumor cells were infected by a GFP-shCD44-2 plasmid or by a GFP-shControl plasmid. As the plasmid did not contain a selection marker, infected cells were distinguished by GFP expression. Infected cells were exposed to TME Stimulation (as in Figure 1). No stimulation = Cells grown with vehicles only. (**A**) CD44 Knock-down validation. CD44 surface expression was determined by FACS analyses, using fluorescently-labeled specific Abs. Isotype = Isotype-matched Abs used as control. (A1) Non-stimulated cells. (A2) Cells exposed to TME Stimulation. (**B**) Cell morphology and paxillin localization, detected by specific Abs (purple). Cell nuclei were visualized by Hoechst staining (blue). Cells in which CD44 was knocked-down express GFP (green). Isotype-matched Abs were used as control, in order to determine baseline staining (Data not shown). (B1) Cells infected with the GFP-shControl plasmid. (B2) Cells infected with the GFP-shCD44-2 plasmid. Bar = 10 μm. In all panels, the results are from a representative experiment of *n* > 3, showing similar results. Photos of cell morphology, detected by light microscopy, are included in Supplementary Figure S5.

CD44 is the common denominator of the two sub-populations which are enriched by TME Stimulation: CD44+/β1+ and CD44+/CD24^low/−^. In this set of experiments, we asked if CD44 controls relevant cell functions (such as cell spreading) and whether it regulates the expression of the other two cell surface molecules that characterize CD44+/β1+ and CD44+/CD24^low/−^ cells, namely β1 and CD24. To this end, MCF-7 cells were infected by GFP-shCD44, and the GFP tag was used to identify cells in which CD44 was knocked-down (construct expression was evaluated by GFP expression because the plasmid did not contain a selection marker). FACS analyses of membranous CD44 expression provided a quantitative evidence to high knock-down efficiency achieved by shCD44 (Figure 4A). The Confocal images of Figure 4B show that shControl-expressing TME-stimulated cells had a highly spread phenotype, as can be also seen by light microscopy photos in Supplementary Figure S5 (white arrows). CD44 down-regulation has impaired the ability of TME-stimulated cells to spread, as indicated by the localization of paxillin (which based on our previous study [40] was used to visualize cell extremities; Figure 4B1 *vs.* 4B2, bottom row). In contrast, analyses of non-stimulated cells demonstrated that their morphology was not affected by CD44 knock-down (Figure 4B2 *vs.* 4B1, top row).

**Figure 5:**
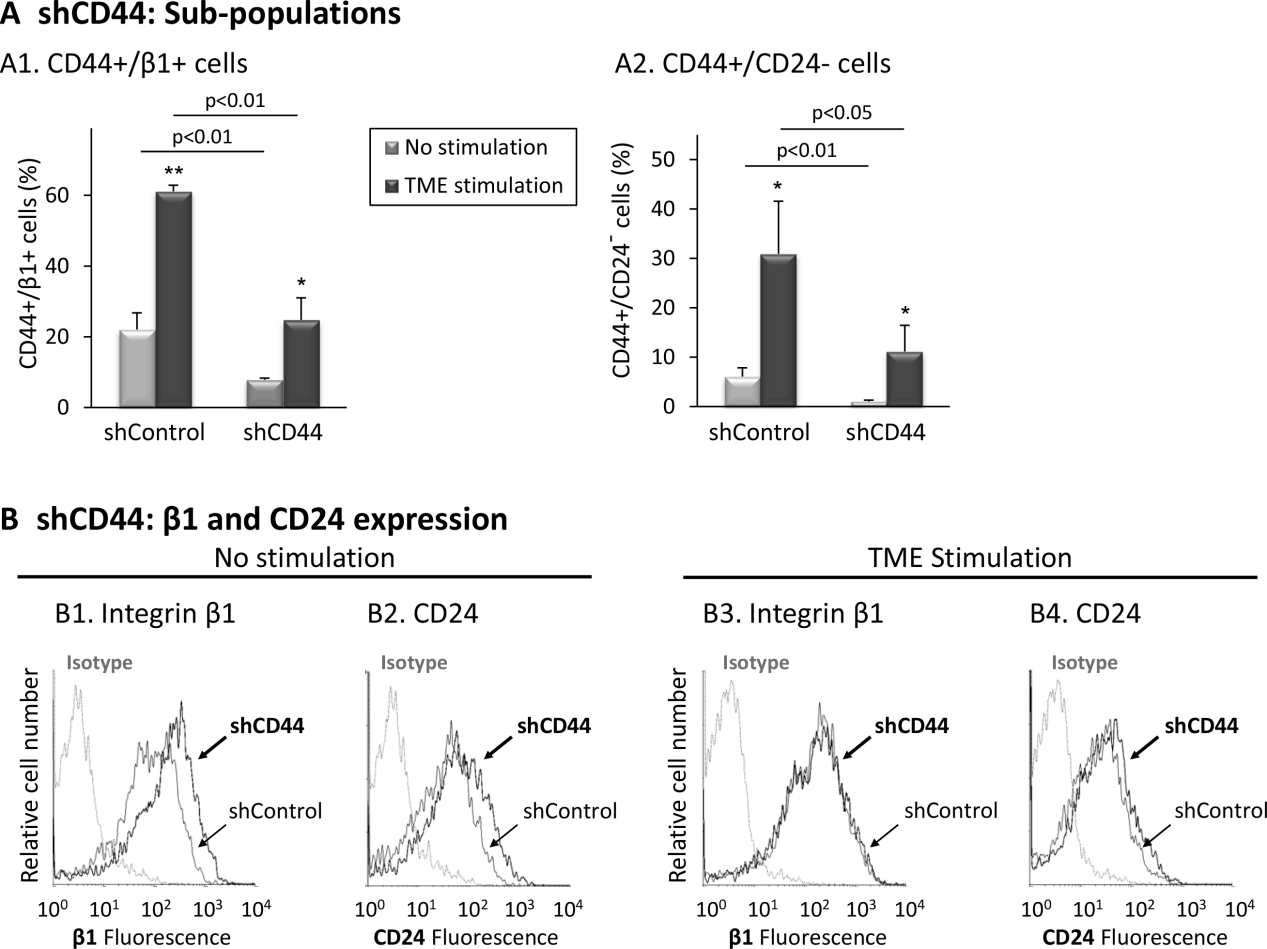
CD44 knock-down reduces the proportions of the TME-enriched CD44+/β1+ and CD44+/CD24- sub-populations Following the procedure described in Figure 4, expression levels of surface markers were determined by FACS analyses, using fluorescently-labeled specific Abs. Isotype = Isotype-matched-Abs used as control. (**A**) Proportions of sub-populations. (A1) CD44+/β1+ cells. (A2) CD44+/CD24- cells [Due to GFP expression, the fluorophores of the Abs used for flow cytometry were replaced, leading to lower CD24 staining signals. As a consequence, the usual setting of CD24 axes gave rise to non-proportionately high levels of CD24^low/−^ cells and were set again to distinguish only CD24- (CD24-negative) cells]. *p < 0.05, **p < 0.01 for differences between TME-stimulated and non-stimulated cells. The panel sums up the results obtained in *n* = 3 independent repeats. A representative experiment of *n* = 3, showing similar results, is demonstrated in Supplementary Figure S6. (**B**) Expression of integrin β1 and CD24. (B1, B2) Non-stimulated cells. (B3, B4) Cells exposed to TME Stimulation. The results are from a representative experiment of *n* = 3, showing similar results. Average expression values ± SD and statistics are provided in Supplementary Figure S6C.

In parallel, we found that as expected, CD44 down-regulation has led to a reduced proportion of the CD44+/β1+ sub-population in the presence and absence of the TME Stimulation (Figure 5A1 and Supplementary Figure S6A). Also, shRNA to CD44 has led to reduced proportions of TME-enriched CD44+/CD24-cells (Figure 5A2 and Supplementary Figure S6B) (here we referred to CD24- only, rather than CD24^low/−^, due to fluorophore-related issues; please see legend to Figure 5A). Single marker analysis (Figure 5B and Supplementary Figure S6C) indicated that CD44 knock-down has led to elevated β1 expression in cells that were not exposed to TME Stimulation (Figure 5B1 and Supplementary Figure S6C). Such β1 up-regulation may reflect a compensatory mechanism for the lack of CD44, enabling the proper cell spreading demonstrated upon CD44 knock-down in these cells (Figure 4B2 *vs.* 4B1, top row). However, upon CD44 knock-down, β1 was not up-regulated in TME-stimulated cells (Figure 5B3), explaining the aberrant spreading of these cells following TME Stimulation (Figure 4B2 *vs.* 4B1, bottom row). Furthermore, in cells which were not stimulated by TME factors, down-regulation of CD44 has up-regulated CD24 expression (though less than β1) (Figure 5B2 and Supplementary Figure S6C). These findings suggest that high levels of CD44 lead to reduced expression of CD24, thus CD44+/CD24^low/−^ cells are generated, identified as CSCs. Together, the findings of Figures 4 and 5 reveal roles for CD44 in regulating cell spreading and generation of the stem-like sub-population in Luminal-A breast tumor cells.

**Figure 6:**
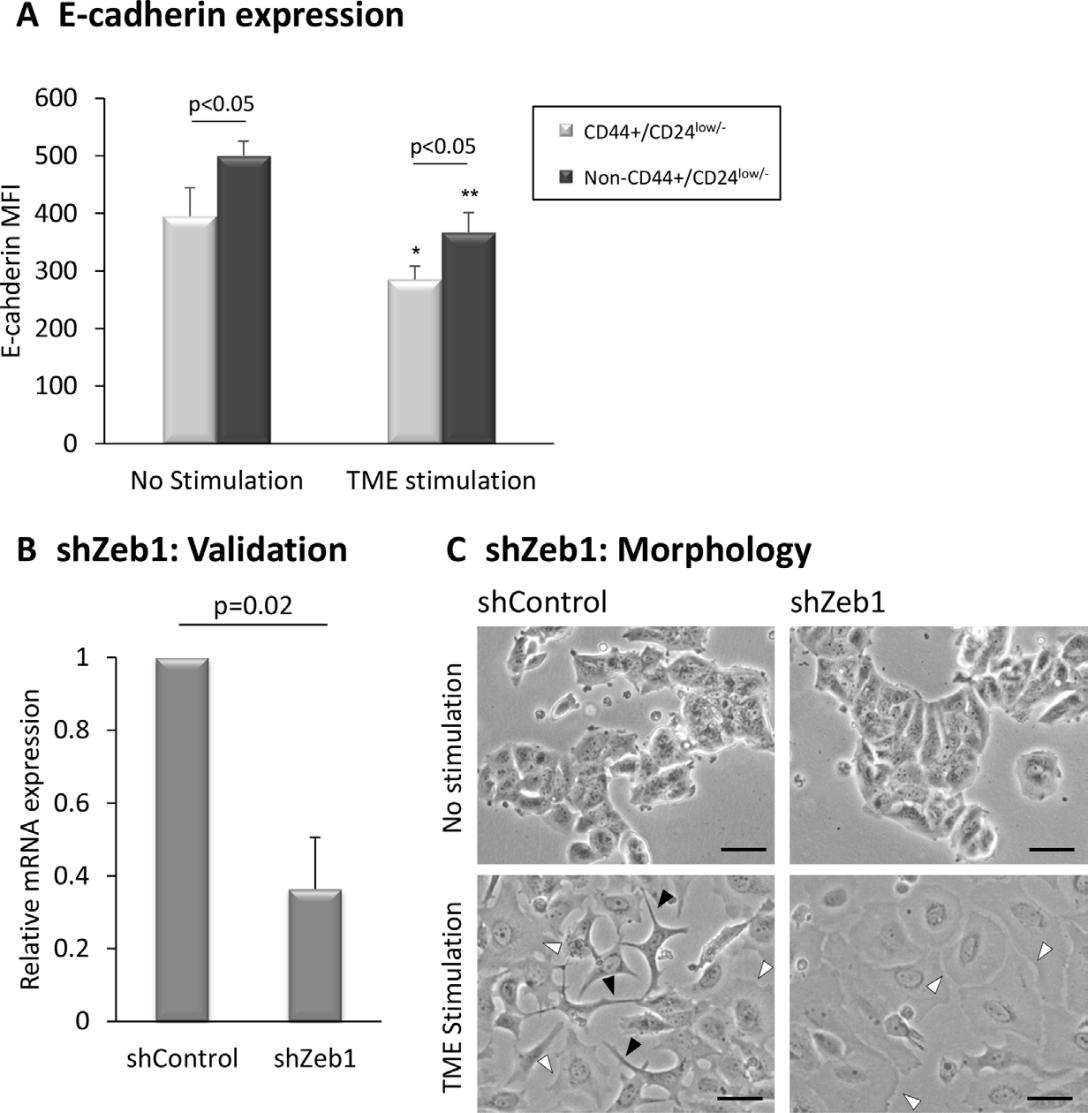
EMT-related characteristics of CD44+/CD24^low/−^ cells (**A**) E-cadherin levels in CD44+/CD24^low/−^ cells. MCF-7 breast tumor cells were exposed to TME Stimulation (as in Figure 1). No stimulation = Cells grown with vehicles only. E-cadherin levels on CD44+/CD24^low/−^ cells were determined by FACS analyses, using fluorescently-labeled specific Abs. *p < 0.05, **p < 0.01 for the difference between TME-stimulated and non-stimulated cells. The panel sums up the results obtained in n>3 independent repeats, demonstrating the E-cadherin MFI (Mean Fluorescence Intensity). (**B**, **C**) Zeb1 knock-down interferes with formation of cellular protrusions. MCF-7 breast tumor cells were infected by a shZeb1 plasmid or by its shControl plasmid and were analyzed following a selection process. (B) Zeb1 Knock-down validation, determined by qPCR analysis at the end of the selection process. shControl-infected cells were given the value of 1. The panel sums up the results obtained in *n* = 3 experimental repeats. (C) Determination of cell morphology by light microscopy. Black arrows: Cells that formed long protrusions and exhibited an EMT-like phenotype. White arrows: Cells that demonstrated extensive spreading abilities, and lack of cellular protrusions. Bar = 50 μm. The results are from a representative experiment of *n* = 3, showing similar results.

Then, to follow on proposed interactions between EMT processes and generation of CSCs [60, 61], we determined whether the CD44+/CD24^low/−^ sub-population expressed typical EMT-related characteristics. Indeed, after (and also prior to) TME Stimulation, CD44+/CD24^low/−^ cells expressed significantly lower E-cadherin levels (Figure 6A) - which is a typical characteristic of cells that have undergone EMT [62–65] - compared to Non-CD44+/CD24^low/−^ cells. These findings support associations between the TME-enriched stem-like cells and acquisition of an EMT-related phenotype by CD44+/CD24^low/−^ cells.

**Figure 7:**
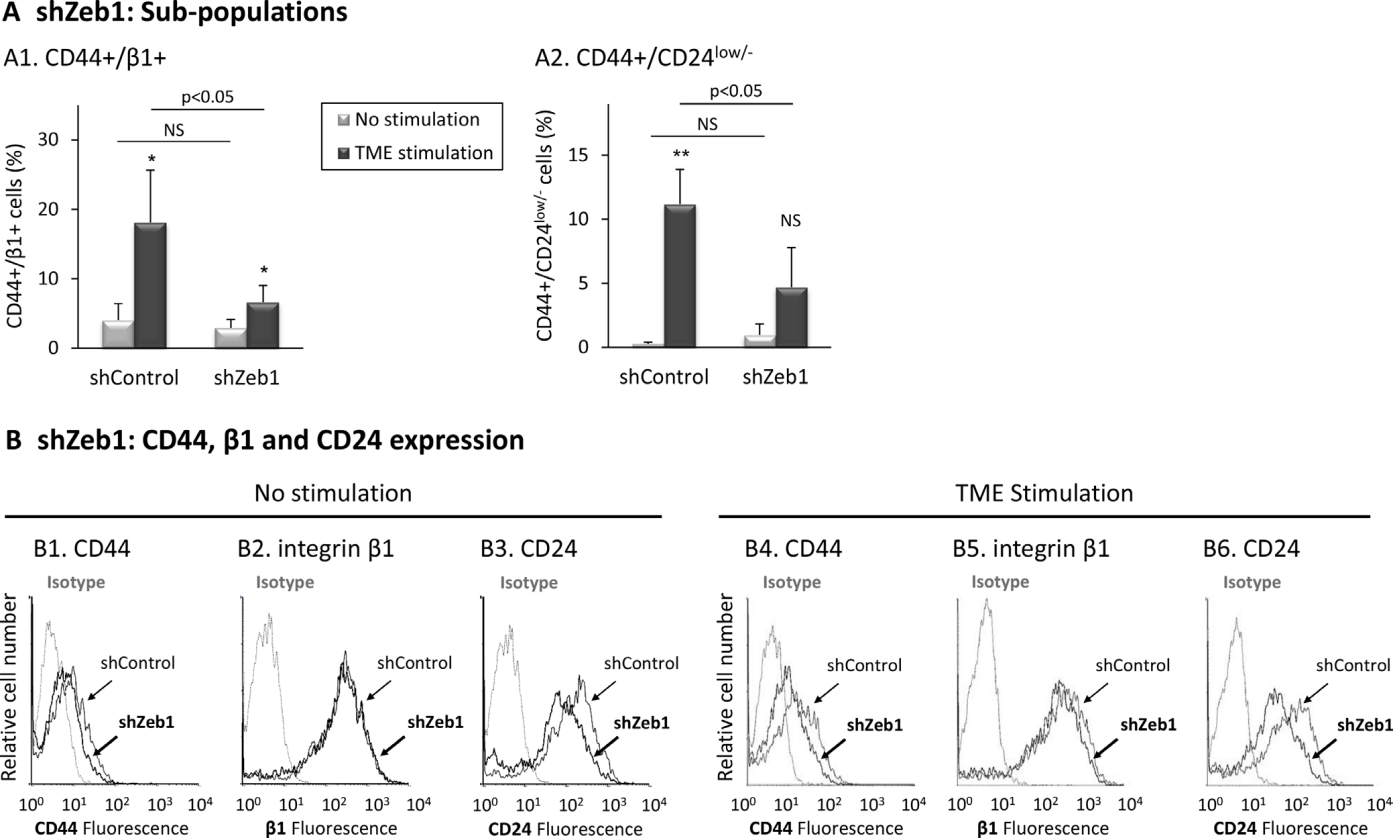
Zeb1 knock-down reduces the proportions of TME-enriched CD44+/β1+ and CD44+/CD24^low/−^ sub-populations Using the same procedure as in Figure 6, expression levels of surface markers were determined by FACS analyses, using fluorescently-labeled specific Abs. Isotype = Isotype-matched Abs used as control. (**A**) Proportions of sub-populations. (A1) CD44+/β1+ cells. (A2) CD44+/CD24^low/−^ cells. *p < 0.05, **p < 0.01 for differences between TME-stimulated and non-stimulated cells. NS = Not significant. The panel sums up the results obtained in *n* > 3 independent repeats. A representative experiment of *n* = 4, showing similar results, is demonstrated in Supplementary Figure 7 (of note, puromycin-induced selection process was employed in order to obtain the desired Zeb1-knocked-down cells, leading to lower proliferation rates in all infected cells, which were accompanied by a reduced enrichment of CD44+/β1+ cells in shControl-infected TME-stimulated cells, compared to Supplementary Figure S1A). (**B**) Expression of CD44, integrin β1 and CD24. (B1–B3) Non-stimulated cells. (B4–B6) Cells exposed to TME Stimulation. The results are from a representative experiment of *n* = 4. Average values ± SD and statistics are provided in Supplementary Figure S7C.

The above findings motivated us to determine whether EMT master regulators control TME-induced morphological changes and enrichment of the CD44+/β1+ and CD44+/CD24^low/−^ sub-populations. To this end, we down-regulated the expression of Zeb1, Snail and Slug, chosen due to their prominent involvement in inducing EMT in different cancers [66–71] and to their roles in generating a TME-Stimulation-induced EMT-like phenotype in Luminal-A breast cancer cells [40]. While knock-down of Snail and Slug yielded inconsistent results in several independent experimental repeats (Data not shown), Zeb1 knock-down has led to pronounced effects (Figures 6 and 7; Supplementary Figure S7). Following validation of knock-down efficiency (Figure 6B), we found that Zeb1 down-regulation reduced considerably the ability of TME-stimulated cells to generate long protrusions and to acquire an EMT-like phenotype (marked by black arrows; Figure 6C, lower right image *vs.* lower left image). In contrast, Zeb1 down-regulation did not affect the ability of TME-stimulated cells to spread (marked by white arrows; Figure 6C). These observations agree with the roles of Zeb1 as a driver of cytoskeletal rearrangement and formation of protrusions in EMT [72–74]. Zeb1 knock-down had little to no impact on the morphology of non-stimulated cells (Figure 6C, upper right image *vs.* upper left image).

In parallel, we found that Zeb1 knock-down has led to marked inhibition in the ability of TME stimulation to enrich for the CD44+/β1+ sub-population (Figure 7A1 and Supplementary Figure S7A), resulting mainly from reduced expression of CD44 in TME-stimulated cells (Figure 7B4 and 7B5; Supplementary Figure S7C). A marked decrease was also noted in the TME-enriched CD44+/CD24^low/−^ sub-population (Figure 7A2 and Supplementary Figure S7B), resulting again predominantly from reduced expression of CD44 (Figure 7B4; Supplementary Figure S7C). Of interest was the fact that CD24, whose reduced levels denote the CSC sub-population, was also reduced by Zeb1 down-regulation in TME-stimulated cells (Figure 7B6; Supplementary Figure S7C).

Overall, the data presented in Figures 4–7 and in Supplementary Figures S6–S7 indicate that CD44 and Zeb1 regulate TME-induced cell spreading and formation of cellular protrusions, although in different manners. In addition, in the absence of TME Stimulation, CD44 was revealed as regulator of expression of its counterpart molecules that mark the CD44+/β1+ and CD44+/CD24^low/−^ sub-populations, β1 and CD24. Most importantly, when the cells were exposed to TME Stimulation, Zeb1 regulated CD44 and CD24 (and to some extent β1), expression, suggesting that EMT-related processes are connected to the generation of TME-induced tumor cellular heterogeneity and generation of CD44+/β1+ and CD44+/CD24^low/−^ sub-populations.

### TME-enriched CD44+/CD24^low/−^ and CD44+/β1+ sub-populations contribute similarly to formation of primary tumors

**Figure 8:**
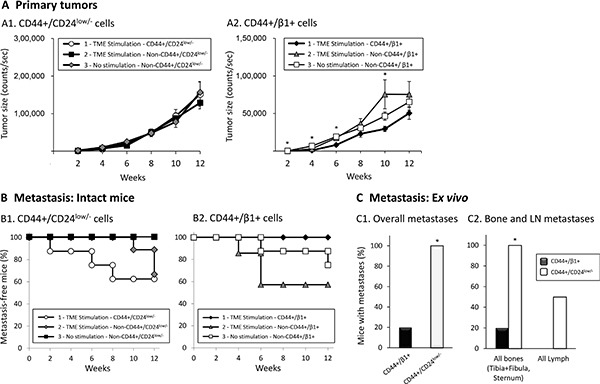
Primary tumors generated following the inoculation of TME-stimulated CD44+/CD24^low/−^ and CD44+/β1+ cells have comparable sizes, but only CD44+/CD24^low/−^ cells promote metastasis formation Development of primary tumors (**A**) and of metastases (**B**, **C**). mCherry- or mPlum-expressing MCF-7 cells were exposed to TME Stimulation for three days in culture (as in Figure 1), labeled by Abs, sorted and inoculated to the mammary fat pad of female mice (5 × 10^3^ live cells/mouse). In the first set of experiments (A1, B1), three types of mCherry-expressing cells were studied: (1) TME-stimulated-CD44+/CD24^low/−^ cells (Group 1); (2) TME-stimulated-Non-CD44+/CD24^low/−^ cells (Group 2); (3) No stimulation-Non-CD44+/CD24^low/−^ cells (Group 3). The same procedure was taken in the second set of experiments (A2, B2), but with mPlum-CD44+/β1+ cells. Statistical analyses were performed as described in “Materials and methods” (where the ability of the different tests to perform pairwise comparisons is indicated). (A) Primary tumors; Average tumor sizes ± SEM are presented as counts/sec of fluorescence emission, obtained at each time point by the CRi Maestro™ intravital imaging system. (A1) At all time points, no significant changes were found between the three groups of mice (*p* > 0.05, ANOVA). (A2) *p < 0.05, ANOVA. (B) Kaplan-Meier analyses of metastasis-free mice, showing incidence of macro-metastases detected by intravital imaging using the CRi Maestro^™^ device, in intact mice. (B1) A significant difference was not expected in this cohort size, yet the differences between the groups were close-to-significant (*p* = 0.087, Log-Rank test). (B2) *p* = 0.231, Log-Rank test. (C) Proportions of mice that developed metastases, detected *ex vivo* by the CRi Maestro™ device at the end of experiments. The excised organs included tumor-adjacent LNs (inguinal), contralateral LNs, leg bones (tibia+fibula), chest bones (sternum+ribs), liver and lungs. (C1) Proportions of mice with metastases in all excised organs. (C2) Proportions of mice with metastases in bones (left) and LNs (right). *p < 0.05 by Fisher's Exact Test, comparing the metastases generated by injections of the two sub-populations. All panels sum up results obtained in *n* = 2 experimental repeats in which tumor cells were inoculated to a total of *n* = 8–11 mice (please see comments on mice numbers and statistics in “Materials and methods”).

At this stage, we evaluated the contribution of each of the TME-enriched sub-populations, CD44+/CD24^low/−^ or CD44+/β1+, to formation of primary tumors (Figure 8A) and to metastatic spread (further described below) of Luminal-A breast tumor cells. Following titration experiments demonstrating that CD44+/CD24^low/−^ formed primary tumors at low cell numbers (e.g., 10^4^cells/mouse) and are thus very aggressive (Supplementary Figure S8), both cell types were injected at 5 × 10^3^ cells/mouse to the mammary fat pad.

Two sets of experiments were performed, each with three groups of mice. The first set consisted of mice inoculated with mCherry-expressing cells, including the following groups: (1) Group 1, termed “TME-stimulated – CD44+/CD24^low/−^“, consisted of CD44+/CD24^low/−^ cells that were sorted out of TME-stimulated cells; This group demonstrates the tumorigenicity phenotype of TME-enriched stem-like cells. (2) Group 2, termed “TME-stimulated – Non-CD44+/CD24^low/−^“, consisted of cells remaining after sorting out the CD44+/CD24^low/−^ cells from TME-stimulated cells; This group demonstrates the tumorigenicity phenotype of all different types of cells that could have been induced by TME Stimulation, albeit without the stem-like cells. (3) Group 3, termed “No stimulation – Non-CD44+/CD24^low/−^“. This group enabled us to compare the tumorigenicity phenotype of all other cell types remaining after removal of CD44+/CD24^low/−^ cells, between TME-stimulated (as in Group 2) and non-stimulated cells (as in Group 3). The second set of experiments was performed in a similar manner, but the tumor cell population that was sorted out was of CD44+/β1+ cells and the cells expressed mPlum. The fluorescent proteins enabled monitoring of primary tumors and metastases by intravital imaging, every two weeks, until the endpoint of experiment, 12 weeks post-inoculation.

The data in Figure 8A1 show that TME-enriched CD44+/CD24^low/−^ cells did not have any advantage over the “remaining” cells (Non-CD44+/CD24^low/−^ cells) in generating primary tumors. Following TME Stimulation and sorting of CD44+/β1+ cells, a modest increase in tumor growth was observed when CD44+/β1+ cells were removed, suggesting that the remaining cells (Group 2) may have a slightly more aggressive phenotype than the CD44+/β1+ cells themselves (Figure 8A2).

### TME-enriched CD44+/CD24^low/−^ cells show an advantage over CD44+/β1+ cells in promoting metastatic dissemination

To follow on the above observations, we determined the ability of each of the two TME-enriched sub-populations, CD44+/CD24^low/−^ and CD44+/β1+ cells, to promote formation of metastases *in vivo*. Intravital imaging demonstrated a highly metastatic behavior for TME-enriched CD44+/CD24^low/−^ stem-like cells (Group 1; Figure 8B1) while the remaining cells, which did not include the CD44+/CD24^low/−^ sub-population, formed metastases at a substantially slower kinetics (Group 2; Figure 8B1).

**Figure 9:**
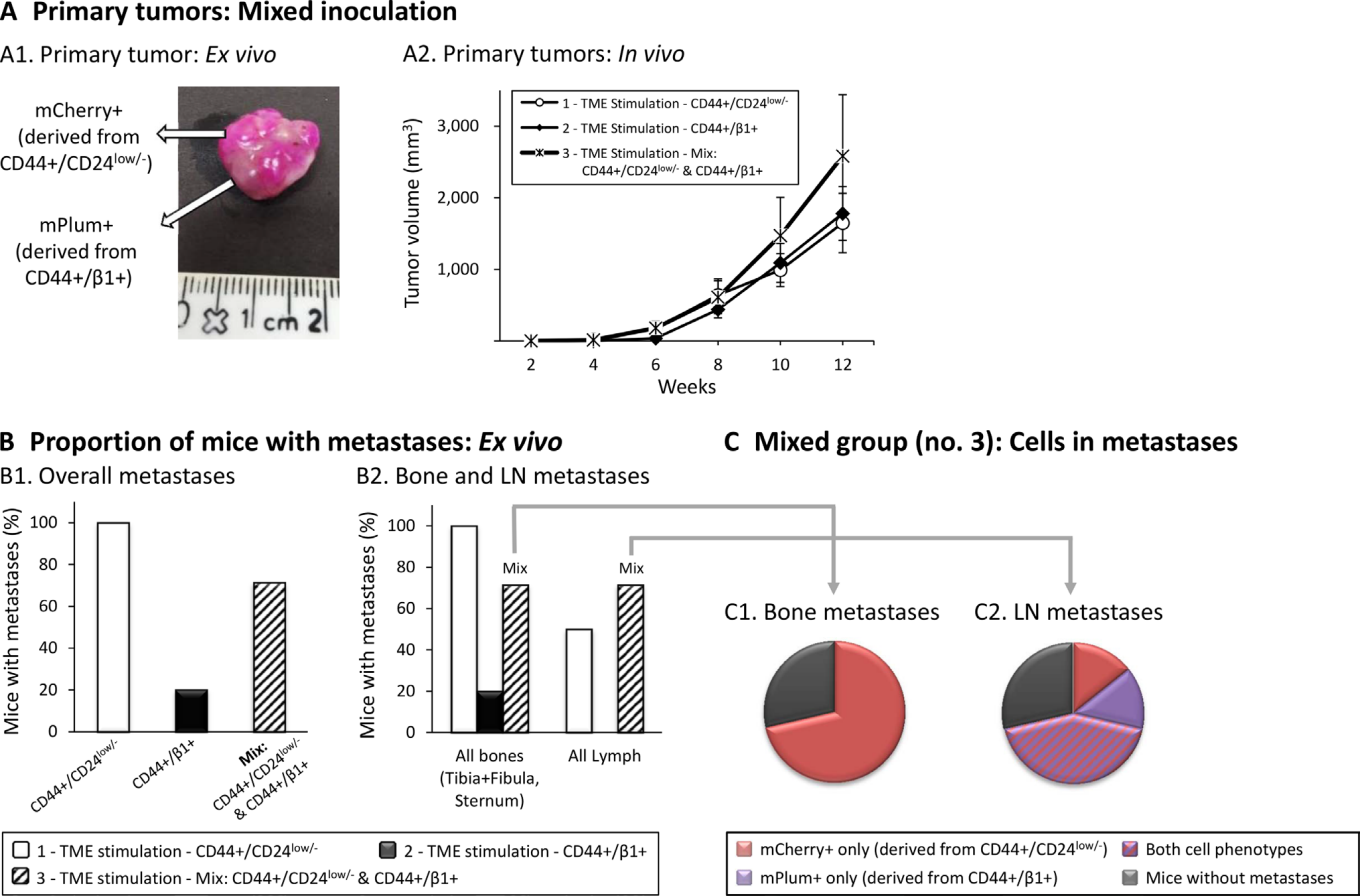
Mixed inoculation of TME-enriched CD44+/CD24^low/−^ and CD44+/β1+ cells further supports the key roles of CD44+/CD24^low/−^ cells in metastasis CD44+/CD24^low/−^ cells and CD44+/β1+ cells were sorted out from TME-Stimulated mCherry- or mPlum-expressing MCF-7 cells, respectively. Three groups of cells were injected (procedure as in Figure 8) to mice: Group 1 - CD44+/CD24^low/−^cells; Group 2 - CD44+/β1+ cells; Group 3 - mixture of both cell types in a 1:1 ratio. In all three groups, the total number of inoculated tumor cells was the same. Primary tumors and metastases were followed for 12 weeks, as described in Figure 8. (**A**) Development of primary tumors. (A1) An image of a resected primary tumor from a mouse of Group 3, demonstrating mCherry-expressing cells derived from sorted CD44+/CD24^low/−^ cells and mPlum-expressing cells derived from sorted CD44+/β1+ cells. (A2) Average sizes of primary tumors ± SEM are presented in mm^3^, followed every two weeks by caliper measurements (*p* > 0.05, ANOVA). (**B**) Metastases detected *ex vivo* by the CRi Maestro™ device. (B1) Proportions of mice that developed metastases in all excised organs. A significant difference was not expected in this cohort size, yet the differences between the three groups were close-to-significant (*p* = 0.056, Fisher's Exact Test). (B2) Proportions of mice that developed metastases in bones (left) and LNs (right). The differences between the three groups for bone metastases were close-to significant (*p* = 0.056, Fisher's Exact Test), and the differences between the three groups for LN metastases were significant (*p* = 0.042, Fisher's exact test). (**C**) Type of metastatic cells found in bones and LNs of mice of Group 3 (inoculated with a 1:1 mix of both sub-populations). (C1) Bone metastases. (C2) LN metastases. All panels sum up results obtained in *n* = 2 experimental repeats in which tumor cells were inoculated to a total of *n* = 8–11 mice (please see comments on mice numbers and statistics in “Materials and methods”).

In contrast to the high metastatic potential of CD44+/CD24^low/−^ cells, the TME-enriched CD44+/β1+ sub-population did not lead to formation of macro-metastases that could be detected by the intravital imaging CRi Maestro™ device (Group 1; Figure 8B2). Although the *in vitro* sorted CD44+/β1+ cells may have contained a certain proportion of CD24^low/−^ cells (and were thus CD44+/β1+/CD24^low/−^ as shown in Figure 2), no metastatic potential was observed when these cells were inoculated to mice. The findings presented further below (Figure 9) suggest that CD44+/β1+/CD24^low/−^ cells, in contrast to CD44+/CD24^low/−^ cells, do not contribute to metastasis or that their proportion was too low to give rise to an overt metastatic process.

In parallel, it was interesting to note that the Non-CD44+/β1+ cells, remaining after sorting out the TME-enriched CD44+/β1 cells, promoted the metastatic phenotype, similar to the sorted TME-enriched CD44+/CD24^low/−^ cells. These findings were expected because after sorting out of CD44+/β1+ cells, the CD44+/CD24^low/−^ sub-population remained and contributed its share to metastasis formation. These findings suggest that when the complete TME-stimulated population is injected to mice (as in [40]), formation of metastases is driven mostly by the CD44+/CD24^low/−^ cells while CD44+/β1+ cells do not have much role in dissemination to remote organs (due to the *in vivo* drift, demonstrated for example in Table 1B, we assume that not all cells that have reached the metastatic sites still retain their original phenotype). These findings identify an aggressive phenotype for the TME-enriched CD44+/CD24^low/−^ sub-population, further supporting their stem-like nature.

At the end of these *in vivo* experiments, organs that are considered favorable metastatic sites were excised and metastasis formation was analyzed *ex vivo* by the CRi Maestro− device, detecting smaller metastatic lesions than intravital analyses. The *ex vivo* findings presented in Figure 8C1 are in line with the intravital results, demonstrating that TME-enriched stem-like CD44+/CD24^low/−^ cells have led to a higher metastatic phenotype than TME-enriched CD44+/β1+ cells. Figure 8C2 further breaks-down the data and demonstrates the substantial advantage of CD44+/CD24^low/−^ cells, promoting bone and LN metastases formation, compared to CD44+/β1+ cells.

The above analyses identified the metastatic potential of each of the two TME-enriched sub-populations, CD44+/CD24^low/−^ cells *vs.* CD44+/β1+ cells, when each was inoculated alone. However, when the entire MCF-7 cell population undergoes the process of TME Stimulation, the two cell populations are enriched simultaneously; thus, they may affect each other's activities. To determine the contribution of each sub-population to metastasis in a heterogeneous setting that includes them both, we conducted experiments comparing each of the two cell sub-populations alone, to a mixture of both. This third set of experiments contained three groups of mice: (1) mCherry-expressing CD44+/CD24^low/−^ cells, sorted out of TME-stimulated cells; (2) mPlum-expressing CD44+/β1+ cells, sorted out of TME-stimulated cells; (3) “TME Stimulation - Mix” - Co-inoculation of both sub-populations (mCherry-CD44+/CD24^low/−^ cells + mPlum-CD44+/β1+ cells, in 1:1 ratio). The total number of cells inoculated to mice, in each group, was identical (5x10^3^ cells/mouse).

The image of Figure 9A1 shows that in mice of Group 3 (inoculated with a mix of the two sub-populations), the primary tumor is co-populated by cells that have originated from both cell types; however, the joint presence of the two sub-populations together did not confer a significant advantage to the cells, over inoculation of each of the sub-populations alone, in generating primary tumors (Figure 9A2). Nevertheless, when metastasis formation was assessed, a different pattern was revealed. Mice inoculated with a mix of both sub-populations (Group 3) demonstrated an intermediate phenotype: they developed less metastases than mice inoculated by the metastatic CD44+/CD24^low/−^ cells (Group 1) and more metastases than mice inoculated by the poorly-metastatic CD44+/β1+ cells (Group 2) (Figure 9B1). These findings suggest that CD44+/β1+ cells “dilute” the content of metastatic CD44+/CD24^low/−^ cells, leading to an overall reduced metastatic capabilities when the two sub-populations co-exist.

Then, when we examined the metastatic development in specific distant organs, interesting trends were observed. When bone metastases were in question, mice inoculated with mixed sub-populations (Group 3) exhibited again an intermediate metastatic phenotype, developing less metastases than mice of Group 1 (CD44+/CD24^low/−^) but more metastases than mice of Group 2 (CD44+/β1+) (Figure 9B2, left panel). When LN metastasis was determined, the tendency in Group 3 was towards higher levels of metastasis than in Group 1 (CD44+/CD24^low/−^), and was certainly higher than in Group 2 (CD44+/β1+) where no LN metastases were observed at all (Figure 9B2, right panel).

To follow on these observations, we further characterized the metastatic spread in mice of Group 3, inoculated with mixed sub-populations. To this end, *ex vivo* analyses were performed with the CRi Maestro− device, taking advantage of the fact that the cells derived from each of the two sub-populations could be distinguished by their distinct markers: mCherry for CD44+/CD24^low/−^-derived cells and mPlum for CD44+/β1+-derived cells. The pie charts summarizing the results obtained in mice injected with mixed populations (Figure 9C) indicate that only cells derived from the CD44+/CD24^low/−^ sub-population were able to reach the bones (Figure 9C1). In contrast, in the LNs, cells that originated from both sub-populations were detected (Figure 9C2). This finding could explain the high proportion of mice with LN metastases that was demonstrated in mice inoculated with “mixed” cells (Group 3; Figure 9B2, right panel). These results are intriguing, as they propose that when CD44+/β1+ cells are inoculated alone their progeny fail to reach the LNs but they can do so when the cells of the stem-like sub-population are present nearby.

To summarize, not only do TME-enriched CD44+/CD24^low/−^ stem-like cells show an advantage over CD44+/β1+ cells in promoting bone and LN metastasis, but CD44+/CD24^low/−^ cells may also assist cells derived of other sub-populations to disseminate to LNs (but not to bones). These findings further emphasize the detrimental role of the TME in selecting for stem-like cells that promote the metastatic spread of Luminal-A breast tumor cells.

## DISCUSSION

In this study, we provide novel evidence to the ability of the TME to regulate intra-tumoral heterogeneity in Luminal-A tumors and to enrich the cancer cells with two sub-populations: CD44+/CD24^low/−^ cells with a stem-like phenotype and CD44+/β1+ cells that express high levels of adhesion molecules. The findings of our study shed light on the metastatic potential of different TME-enriched sub-populations, showing that disseminating abilities are endowed strongly to CD44+/CD24^low/−^ cells while CD44+/β1+ cells are hardly metastatic. Furthermore, our findings suggest that when the two sub-populations co-exist in tumors, the metastatic potential of CD44+/CD24^low/−^ stem-like cells may not come into full power because they are “diluted” by cells that have low metastatic capabilities, like the CD44+/β1+ cells.

In line with other studies [56, 75], the CD44+/CD24^low/−^ sub-population comprised only ~1% of the original Luminal-A breast tumor cells, but we demonstrated that the proportion of these cells was profoundly increased by TME Stimulation (to ~15% or ~5%, in MCF-7 and T47D cells, respectively). These findings provide important evidence to the power of the TME in enriching stem-like cells and to its key roles in selecting cells with a high metastatic potential. This highly invasive phenotype is remarkable as the cells were exposed to factors of the TME for only three days in culture prior to their inoculation to mice, and enrichment of highly metastatic stem-like cells did not require any genetic manipulation (as done by others [76]).

The data presented in this study indicated that the stem-like sub-population, characterized by the CD44+/CD24^low/−^ phenotype is the major contributor to bone and lymph node metastasis in Luminal-A breast tumors. To date, not many studies have addressed the correlation between the CD44+/CD24^low/−^ sub-population and disease progression in breast cancer patients, and only few of the studies analyzed patients diagnosed with different subtypes of disease. The reports employed different endpoints and often used small patient cohorts, and eventually reached different conclusions. In this context, Balic *et al*. demonstrated that the majority of bone marrow patient samples that included disseminated tumor cells expressed the putative CD44+/CD24- stem cell phenotype [77]. Other studies indicated that the percentage of CD44+/CD24- cells is increased in lymph node metastases in invasive ductal carcinoma and that there is a high correlation between the presence of cells expressing CSC markers and metastases in regional lymph nodes [78, 79]

Specifically reagrding the corrleation between the CD44+/CD24^low/^ sub-population and metastatic dissemination in Luminal-A breast cancer patients, not many reports are available. Within the different reports, the study by Chekhun and colleagues [78] did not find signficant correlations between the CD44+/CD24− sub-population and survival in Luminal-A patients. On the other hand, the study by Lee *et al.* [80] revealed significant association of the ALDH1+/CD44+/CD24- sub-population with Luminal-A tumors and proposed that the CD44+/CD24− phenotype is related to HER2+ tumors putatively originating from luminal-committed progenitors. Moreover, the study by Tsunoda *et al.* demonstrated that tumors of the CD44+CD24^−/low^ type were signficantly associated with axillary lymph node metastasis in luminal tumors [81]. These findings add to a previous study demonstrating that the prevelance of CD44+/CD24^low/−^ cells favors distant metastasis in breast cancer [82], further supporting the potential clinical relevance of our study.

The yet undefined origins of CSCs require intensive characterization in many different research systems. The findings of our study suggest that when CSCs are generated in response to TME factors, the “plastic model” of CSC generation may indeed be valid. Specifically, we have demonstrated that stem-like cells could be selected out of fully differentiated Luminal-A breast tumor cells and that the cells that have been derived following exposure to TME Stimulation were plastic and could undergo a phenotypic drift *in vitro* and *in vivo.*

In parallel, the non-metastatic nature of the CD44+/β1+ requires consideration. Published studies demonstrated increased invasive properties for CD44+ [44–46] or for β1+ cells [42, 43], suggesting that increased adhesion is required for optimal extravasation to remote organs. While our findings support the involvement of TME-induced CD44 and β1 expression in cell adhesion and spreading (Figures 4 and 6), they suggest that elevated expression of both surface molecules simultaneously leads to excessive adhesion, due to which the tumor cells cannot efficiently detach and intravasate the circulation or lymphatics. More detailed analyses of the CD44+/β1+ sub-population may provide improved information on their functional phenotype, as would also be the case for the CD44+/CD24^low/−^ sub-population. Along these lines, our preliminary findings (Data not shown) indicate that the expression of the angiogenic and pro-inflammatory chemokine CXCL8 is higher in TME-enriched CD44+/β1+ cells than in Non-CD44+/β1+ cells, and also in TME-enriched CD44+/CD24^low/−^ cells compared to Non-CD44+/CD24^low/−^ cells. These findings propose that the CD44+/β1+ and also the CD44+/CD24^low/−^ sub-populations that were enriched by TME Stimulation can both demonsrate elevated pro-angiogenic activities. However, additional preliminary findings suggested that different pro-tumorigenic genes may be regulted differently than CXCL8. For example, the expression of CCL2 which is predominantely involved in breast cancer progression, was reduced in the CD44+/β1+ sub-population compared to Non- CD44+/β1+ cells (Data not shown). This finding may provide a partial explanation as to the low metastatic potential of these cells, and calls upon a more systematic future analyses of the expression profiles of the CD44+/CD24^low/−^ and of the CD44+/β1+ sub-populations, that would provide more comprehensive insights to their functions.

Our study also revealed that the stem-like sub-population of CD44+/CD24^low/−^ cells has a typical EMT characteristic as illustrated by reduced E-cadherin expression levels. We also found that the EMT-regulator Zeb1 was required for the generation of such stem-like cells. These findings add to previous studies on the associations between the CSC state and the EMT process, and on published studies by Weinberg and colleagues, demonstrating that Zeb1 is required for CSC activity and enables CD44^low^-to-CD44^high^ conversions [60]. Furthermore, our findings indicate that CD44 and Zeb1 regulate the expression levels of β1 and CD24 and extend published observations on the associations between Zeb1 and CD44 [60, 61]; however, we also found that CD44 and Zeb1 have divergent roles in controlling cell spreading and formation of protrusions: while CD44 is required for cell spreading, Zeb1 is essential for formation of long cellular protrusions. Thus, by elevating both CD44 and Zeb1 expression in the cells (demonstrated in our published study [40]), TME Stimulation enriches for mixed cellular sub-populations having different morphological and functional phenotypes. Evidently, such TME activities can give rise to elevated intra-tumoral heterogeneity and select for the metastatic stem-like CD44+/CD24^low/−^ cells.

Our findings suggest that the two widely-used surface markers of CSCs - CD44 and CD24 - can distinguish between stem-like cells having high metastatic abilities and non-stem-like cells. In the past, ALDH1 was suggested as a complementary marker that characterizes breast CSCs [52, 83]. However, recent studies suggest that there are several distinct CSC sub-populations in breast tumors, one is characterized by the CD44+/CD24^low/−^ phenotype, and the other by ALDH1+ [54]. Our observations, showing that TME Stimulation induced CD44+/CD24^low/−^ cells but not ALDH1+ cells, support this hypothesis and suggest that specific combinations of TME factors may favor the generation of the CD44+/CD24^low/−^ CSC sub-population over the ALDH1+ sub-population. Thus, it is possible that different signals arising from the TME lead to co-existence of several stem-like sub-populations in breast tumors.

In all, the findings of this study, describing the roles of TME factors in enriching a metastatic stem-like sub-population in Luminal-A breast tumor cells, may have important clinical implications. Targeting and eliminating CSCs is a challenging task because the markers of this sub-population are not fully identified, and the currently-used markers are shared by other cell types. Instead, it may be more practical to inhibit the axes that lead to the enrichment and generation of CSCs. Our findings provide a subtype-specific identification of such axes, as they demonstrate that the stem-like sup-population of Luminal-A breast tumor cells is enriched by combined stimulation with three TME arms that are typical of luminal tumors: hormonal (represented by estrogen), inflammatory (TNFα) and growth-stimulating (EGF).

Taking the TME-inhibitory approach in order to target CSCs in Luminal-A tumors is well within reach. Anti-estrogen therapies are the treatment-of-choice for patients with luminal tumors [84–86] and inhibitory modalities against TNFα are successfully used to treat inflammatory diseases, and are considered relatively safe [87–89]. Therapies targeting the EGF axis by inhibiting EGFR and HER2 are currently not offered to Luminal-A patients because their tumors do not carry HER2 amplification or over-expression; but our studies suggest that Luminal-A patients may benefit from such therapies, especially if they would be combined with anti-estrogens and anti-inflammatory drugs. Thus, our current study, as well as our previous works on the impact of combined TME Stimulation on Luminal-A breast tumors [40, 41], suggests that the combination-approach in which several TME factors are to be targeted simultaneously, is very relevant for this subtype of disease. Such a strategy may offer an added value to therapies offered to Luminal-A patients, as it may prevent the enrichment of the CSC sub-population with its devastating contribution to metastasis and resistance to chemotherapies.

## MATERIALS AND METHODS

### Cell cultures

In this research we studied two well-established Luminal-A cell lines of human breast tumors: MCF-7 and T47D cells [50, 90, 91]. MCF-7 cells were kindly provided by Dr. Kaye (Weizmann Institute of Science, Rehovot, Israel) and were authenticated as previously described [40]. T47D cells (clone 11 [92]) were provided by the researcher who generated this cell line, Dr. Keydar (Tel Aviv University, Tel Aviv, Israel) [93]. The two cell lines were grown in culture as previously described [40].

### Cell exposure to TME stimulation

MCF-7 and T47D cells were grown and stimulated as previously described [40]. Briefly, the cells were exposed in culture for three days to “TME Stimulation”, consisting simultaneously of 10^−8^ M estrogen (#E8875; Sigma, Saint Louis, MO), 50 ng/ml TNFα (#300-01A; PeproTech, Rocky Hill, NJ) and 30 ng/ml EGF (#236-EG; R&D systems, Minneapolis, MN) [Stimulation conditions were selected based on titration and kinetics analyses (Data not shown) and agree with those of other publications]. Stimulation was performed in phenol red-free and serum-free DMEM (#01-055; Biological Industries, Beit Haemek, Israel). Medium, including the stimulators, was changed daily. In all procedures, control non-stimulated cells were grown in the presence of vehicles, namely diluents of the stimulating factors [ethanol; 0.1% bovine serum albumin (BSA) and 10 mM acetic acid diluted in double distilled water].

When applicable, cells were also exposed to 0.1 μM doxorubicin (Teva Pharmaceutical, Netanya, Israel; Kindly provided by Dr. Peer, Tel Aviv University). Concentration was selected based on titration analyses (Data not shown).

### Retroviral infections: over-expression of fluorescent proteins (mCherry or mPlum)

To generate mCherry- or mPlum-expressing MCF-7 breast tumor cells, retroviral infections were performed as previously described [40]. Briefly, the mCherry-pQCXI or mPlum-pQCXI plasmids (each with a puromycin selection marker) were transfected by calcium phosphate to HEK293T cells (a generous gift from Dr. Bacharach, Tel Aviv University) together with plasmids encoding gag-pol and VSV-G proteins. Supernatants were collected after two days, filtered through a 0.45-μm mesh and incubated with MCF-7 cells in the presence of 8 μg/ml polybrene for 5 hrs. The infection process was repeated on the following day, in order to increase infection yields. Three days following the second infection, infected cells were selected in 8 μg/ml puromycin (#P-1033; A.G. Scientific, San Diego, CA) for seven days.

### Lentiviral infections: knock-down of CD44 or Zeb1

HEK293T cells were transfected by calcium phosphate with 10 μg lentiviral pRRL GFP-shCD44-2 plasmid or the control pRRL GFP-shLuciferease plasmid (both not containing selection markers; Addgene plasmids 19123 and 19125, respectively, created in the lab of Dr. Weinberg [94]). Alternatively, 10 μg lentiviral pLKO.1-shZeb1 plasmid (#RHS4533-EG6935, clone TRCN0000017566, GE Healthcare Dharmacon, Buckinghamshire, UK) or its control non-coding shRNA pLKO.1 plasmid (#SHC002; Sigma) (both containing puromycin selection marker), were used. In parallel, the cells were transfected by 10 μg of plasmids encoding pCMV-Δ8.2 and VSV-G proteins. Supernatants were collected after two days, filtered through a 0.45-μm mesh (in the process of CD44 knock-down they were diluted 1:7) and incubated with MCF-7 cells in the presence of 8 μg/ml polybrene for 5 hrs. shCD44-infected cells were identified by GFP expression, three-four days after the infection process (no selection process could be performed, since these plasmids lacked a selection marker). For Zeb1 knock-down, the infection process was repeated on the following day to increase infection yield and three days following the second infection, selection was induced by adding puromycin (as above). Then, the cells were plated for different experimental procedures.

### Flow cytometry

Membranous expression levels of cell surface molecules was determined by flow cytometry (FACS) using a Becton Dickinson FACSort (BD Biosciences, San Jose, CA). The following antibodies (Abs) were used: Phycoerythrin (PE)-conjugated mouse IgG1 against integrin β1 (#303004; Biolegend, San Diego, CA); Alexa 488-conjugated rat IgG2b against CD44 (#103015; Biolegend); Alexa 647-conjugated rat IgG2b against CD44 (#103017; Biolegend); PE-conjugated mouse IgG2a against CD24 (#555428; BD Biosciences, Franklin Lakes, NJ); Alexa 647-conjugated mouse IgG2a against CD24 (#311109; Biolegend); mouse IgG1 against E-cadherin (#324101; Biolegend), followed by DyLight 649-conjugated Goat anti-mouse IgG (#405312, Biolegend). Baseline staining was determined by non-relevant isotype-matched Abs as controls. To define CD24^low/−^ cells, CD44/CD24 dot blot analysis of non-stimulated cells was used, in which the axis of CD24 was set just below the cluster of CD44+/CD24+ cells. All cells below this axis were considered CD24^low/−^. An exception was made in GFP-expressing cells, were the axes were set as described in the legend to the relevant. Figure ALDH1 activity was determined using the ALDEFLUOR™ kit (#01700; STEMCELL technologies, Vancouver, Canada), using DEAB inhibitor to determine baseline staining (according to the manufacturer's instructions). All staining patterns and analyses were performed using the win MDI software.

### Confocal microscopy

MCF-7 cells were plated on coverslips in 24-well plates and exposed to TME Stimulation (or to vehicle controls) for three days. Then, cells were fixed with 8% paraformaldehyde (#1.04005; Merck KGaA, Darmstadt, Germany), permeabilized by 0.2% Triton (#X-100; Sigma) and blocked with 2% BSA (#0332-TAM; Amresco, Solon, OH). Mouse IgG1 Abs against paxillin (#624001; Biolegend) were followed by Alexa-647-conjugated Abs against mouse IgG (#115-606-146; Jackson Immunoresearch laboratories, West Grove, PA). In parallel, nuclei were visualized by Hoechst 33342 (#B2261; Sigma). Baseline staining was determined by isotype-matched Abs. Coverslips were mounted using fluorescent mounting medium (#E18-18; Golden Bridge International, Mukilteo, WA) and read by Zeiss LSM 510-META confocal microscope (Carl Zeiss, Jena, Germany).

### Quantitative real-time polymerase chain reaction (qRT-PCR)

Total RNA was isolated from MCF-7 cells using the EZ-RNA kit (#20-400-100; Biological Industries), ten days post infection with a Zeb1 knock-down vector. qRT-PCR analyses were performed as previously described [40]. Dissociation curves for each primer set indicated a single product, and no-template controls were negative. Quantification was performed by standard curves, on the linear range of quantification.

### *In vivo* mouse models of tumor growth and metastasis

mCherry- or mPlum-expressing MCF-7 breast tumor cells were exposed *in vitro* to TME Stimulation (or to vehicle controls) for three days. Then, cells were stained by fluorescently-labeled Abs against CD44, CD24 and/or integrin β1, as appropriate. mCherry-expressing cells were double-labeled to detect and sort CD44+/CD24^low/−^ cells, while mPlum-expressing cells were double-labeled to detect and sort CD44+/β1+ cells. Cells were sorted using a Becton Dickinson FACSAria (BD Biosciences), under sterile conditions. Sorted cells were centrifuged, resuspended in phosphate-buffered saline, mixed 1:1 with matrigel (#356234; BD Biosciences) and 5 × 10^3^ live cells were inoculated orthotopically to the mammary fat pad of 6–8 weeks old female athymic nude mice (Harlan Laboratories, Jerusalem, Israel). One week prior to tumor cell inoculation, mice were implanted subcutaneously with slow-release estrogen pellets (1.7 mg/pellet, 90-days release; #NE-121; Innovative Research of America, Sarasota, FL). The CRi Maestro™ or the IVIS imaging systems were used to monitor tumor growth and metastasis formation in intact mice. At the endpoint of experiments, 12 weeks post-inoculation, mice were euthanized and metastases were detected in excised organs *ex vivo* by the CRi Maestro™ device. The excised organs included tumor-adjacent LNs (inguinal), contralateral LNs, leg bones (tibia+fibula), chest bones (sternum+ribs), liver and lungs. For each type of experiment (with cells sorted for CD44+/CD24^low/−^ cells or for CD44+/β1+ cells or “mix” of both) two independent repeats were performed, inoculating a total of 8–11 mice/group. In the course of the experiments, some of the mice died before the endpoint of experiments (mainly in the group inoculated with the most aggressive CD44+/CD24^low/−^ sub-population) and were not included in the *ex vivo* analyses (performed with *n* = 4).

All procedures involving experimental animals were approved by Tel Aviv University Ethics Committee (Approval #L-14-014), and were performed in compliance with local animal welfare laws, guidelines and policies. The regulations of Tel Aviv University Animal Care Committee did not allow continuation of the experiments to the stage of survival analysis.

### Dissociation of murine tumors

At the endpoint of *in vivo* studies, mice were euthanized, and their primary tumors were resected. The tumors were immediately submerged into ice-cold Hank's Balanced Salt Solution (#02-018; Biological Industries), and then minced into 2-4 mm fragments. Tumor fragments were incubated for 1 hr at 37°C in a mechanical shaker in a dissociation solution containing 1 mg/ml collagenase type IV (#C5138), 20 U/ml DNase type IV (#D5025) and 0.1 mg/ml hyaluronidase type V (#6254) (all from Sigma). The solution with the tumor fragments was filtered through a 70-μm nylon mesh cell strainer and centrifuged. After lysis of erythrocytes by hypotonic water shock, cells were washed and labeled for the expression of CD44, CD24 and/or β1, as appropriate, by FACS analyses. To specifically analyze tumor cells, gating was performed on cells positive for mCherry or mPlum expression, as appropriate.

### Data presentation and statistical analyses

Experiments are presented as means ± standard deviation (SD) or standard error of mean (SEM), as indicated in figure legends. Results were compared by two-tailed unpaired Student's *t*-test or one-way ANOVA. Proportions of metastases-bearing mice were compared by Fisher's Exact test. Kaplan-Meier curves were analyzed by log-rank test using SPSS software (version 22; SPSS Inc., Chicago, IL). Statistical analyses that compared data of > 2 groups could generate only a single *p*-value and could not provide pairwise comparisons between two specific groups. *p* < 0.05 values were considered statistically significant.

## SUPPLEMENTARY MATERIALS



## Abbreviations

Abs: Antibodies
BSA: Bovine Serum Albumin
CSCs: Cancer Stem Cells
ECM: Extracellular Matrix
EGF: Epidermal Growth Factor
EMT: Epithelial-to-Mesenchymal Transition
ER: Estrogen Receptor
Flow: Cytometry
FACS: Fluorescence-Activated Cell Sorting
GFP: Green Fluorescent Protein
LNs: Lymph Nodes
PE: Phycoerythrin
SD: Standard Deviation
SEM: Standard Error of Mean
shRNA: Short Hairpin Ribo-Nucleic Acid
TME: Tumor Microenvironment
TN: Triple Negative
TNFα: Tumor Necrosis Factor α
