# Supplementary material for: Microenvironmental networks promote tumor heterogeneity and enrich for metastatic cancer stem-like cells in Luminal-A breast tumor cells
Source: Oncotarget. 2016 Nov 8;7(49):81123–43. doi: 10.18632/oncotarget.13213 (PMC5348381; doi:10.18632/oncotarget.13213)
Supplement: Supplementary file 1 [file oncotarget-07-81123-s001.pdf]

Microenvironmental networks promote tumor heterogeneity and enrich for metastatic cancer stem-like cells in Luminal-A breast tumor cells

Supplementary Materials

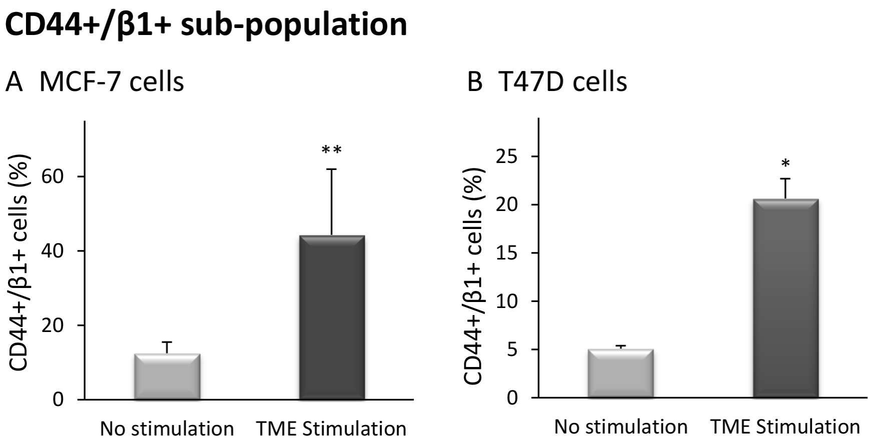

Supplementary Figure S1: TME Stimulation enriches for the CD44+/β1+ sub-population in MCF-7 and T47D breast tumor cells. The figure sums up the proportions of cells obtained following TME Stimulation in $n \geq 3$ independent experimental repeats. The corresponding dot plots were presented in our published manuscripts [40, 41] and their summary is provided herein for the sake of clarity. (**A**) MCF-7 cells. (**B**) T47D cells. $*p < 0.05$, $**p < 0.01$ for the difference between TME-stimulated and non-stimulated cells.

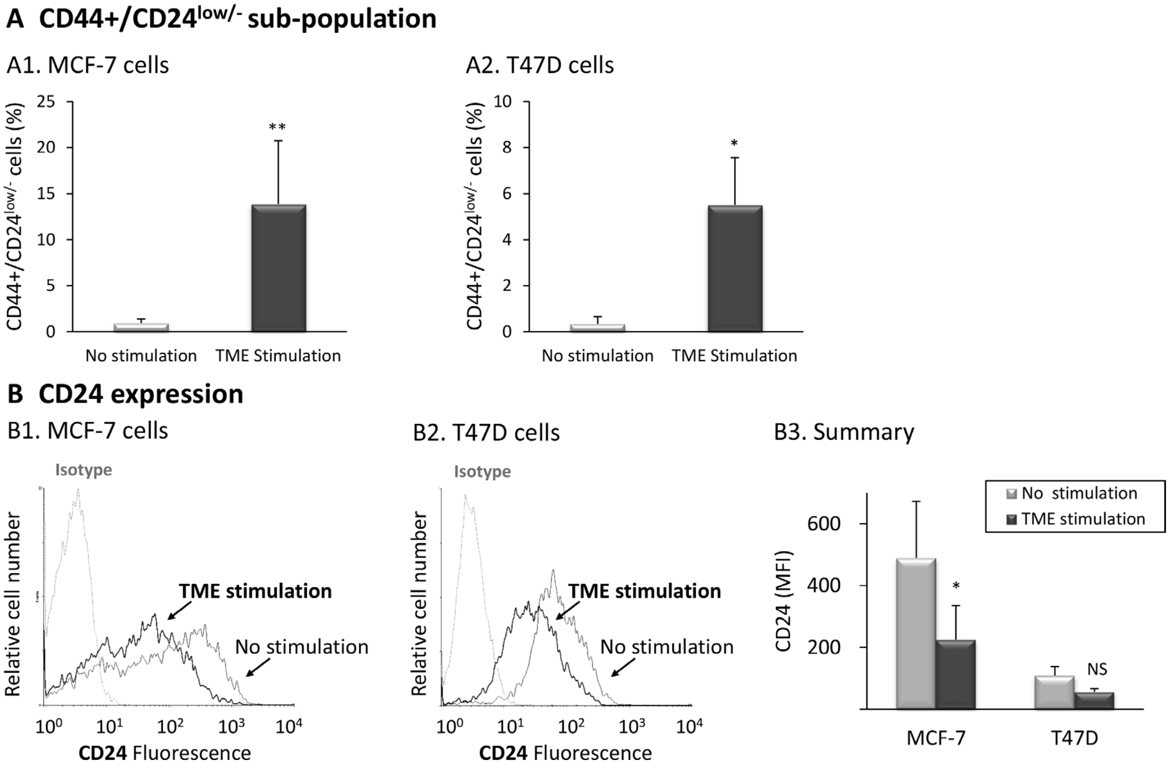

Supplementary Figure S2: TME Stimulation enriches for the CD44+/CD24$^{low/-}$ sub-population in MCF-7 and T47D breast tumor cells. (**A**) The panel sums up the proportions of CD44+/CD24$^{low/-}$ cells obtained following TME Stimulation in $n \geq 3$ independent experimental repeats, whose representative experiments are demonstrated in Figure 1. (A1) MCF-7 cells. (A2) T47D cells. *$p < 0.05$, **$p < 0.01$ for the difference between TME-stimulated and non-stimulated cells. No stimulation = Cells grown with vehicles only. (**B**) CD24 membranous expression. Cells were exposed to TME Stimulation and stained for CD24 expression as in Figure 1. Isotype = Isotype-matched Abs used as control. (B1) MCF-7 cells. (B2) T47D cells. (B3) Summary of CD24 MFI (Mean Fluorescence Intensity) values, obtained in $n \geq 3$ independent experimental repeats. In the case of T47D cells, all experimental repeats demonstrated the same trend but to differing extents. Therefore, despite the similar trend of the results, they were not statistically significant (NS).

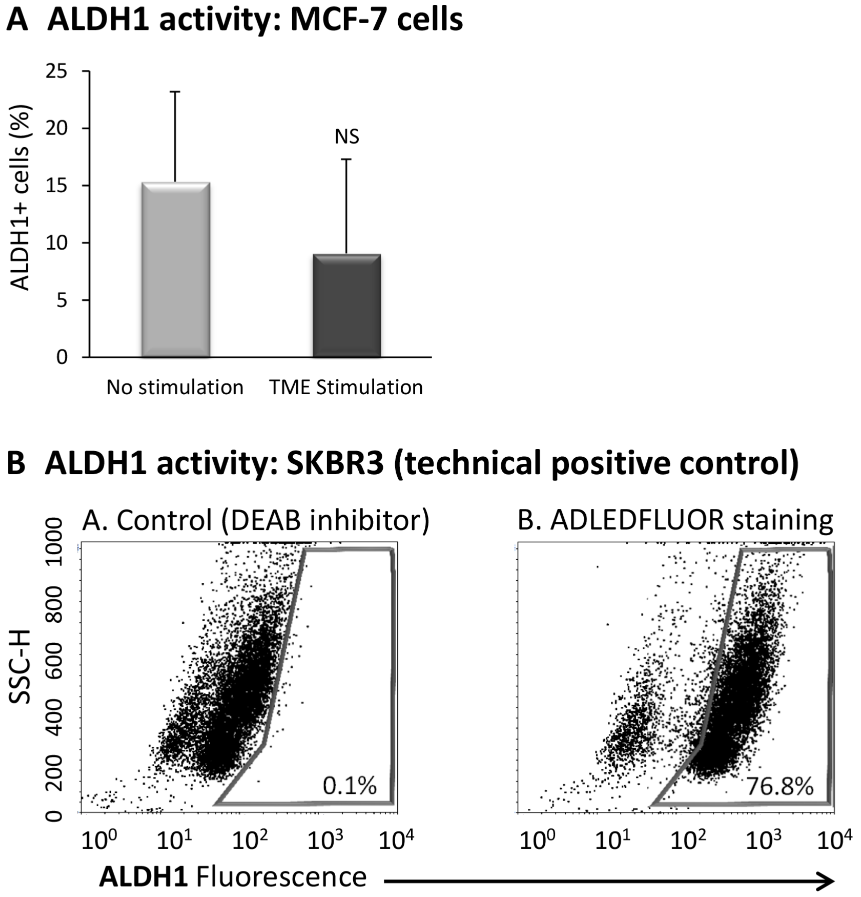

Supplementary Figure S3: TME Stimulation does not enrich for ALDH1+ cells. (**A**) The panel sums up the results of $n > 3$ experiments determining ALDH1 activity in MCF-7 cells, whose representative experiment is presented in Figure 3A. NS = Not Significant. (**B**) ALDH1 activity in SKBR3 cells, serving as positive control for ALDEFLUOR assay. The results were obtained in one of the $n > 3$ experimental repeats performed with MCF-7 cells (Figure 3A and Supplementary Figure 3A), in which SKBR3 cells were also included.

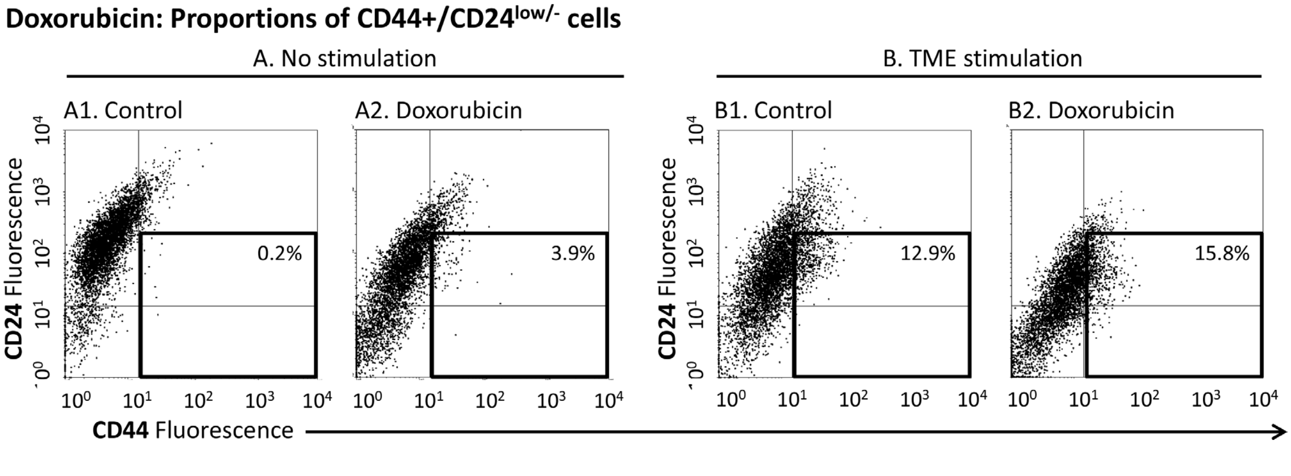

Supplementary Figure S4: Doxorubicin enriches the proportion of the CD44+/CD24$^{low/-}$ sub-population in TME-stimulated cells. The figure demonstrates a representative experiment of $n = 3$, whose summary is presented in Figure 3B. In brief, following exposure to TME Stimulation and doxorubicin, the proportions of CD44+/CD24$^{low/}$ cells were determined by FACS analyses. (**A**) Non-stimulated cells, not exposed (A1) or exposed (A2) to doxorubicin. (**B**) TME-stimulated cells, not exposed (B1) or exposed (B2) to doxorubicin.

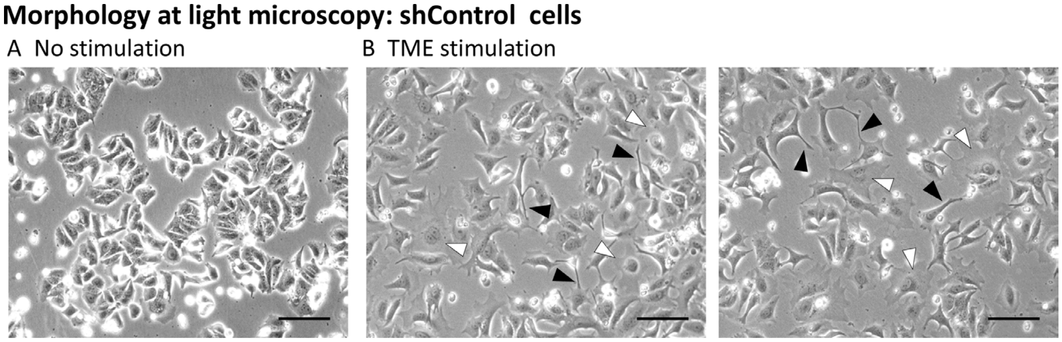

Supplementary Figure S5: Light microscopy photos of MCF-7 cells – Complementing the confocal microscopy images demonstrated in Figure 4B. MCF-7 cells were infected by a shControl plasmid (used as control in Figure 4), in non-stimulated (**A**) and in TME-stimulated cells (**B**) (In part B, two representative photos of the TME-stimulated cells are demonstrated). Cell morphology was determined by light microscopy. Black arrows: Cells that formed protrusions; White arrows: Cells that had an extensive spreading phenotype. Bar = 50 µm. The results are from a representative experiment of *n* > 3, showing similar results.

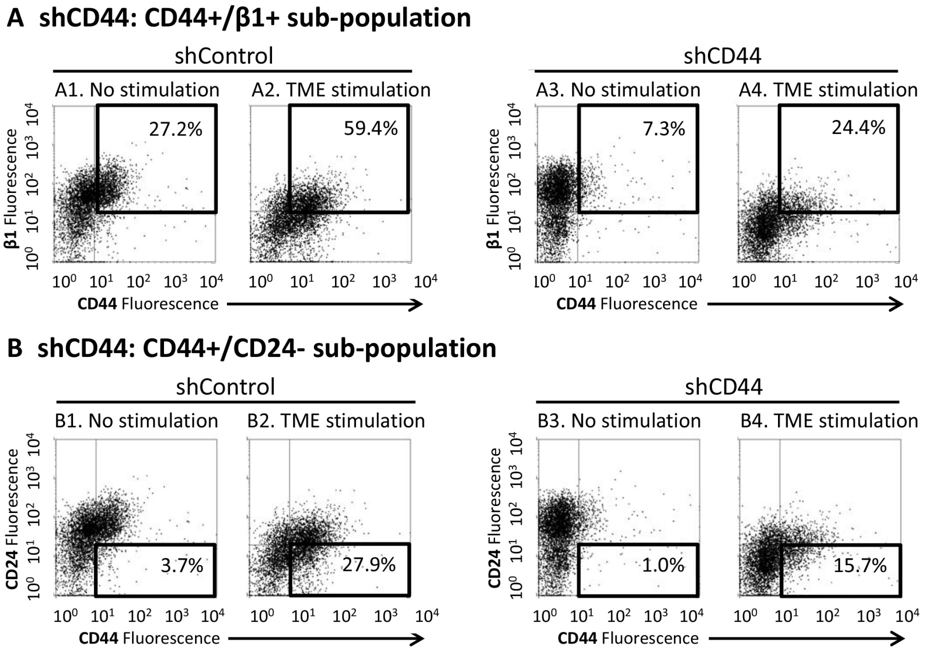

C shCD44: β1 and CD24 expression - Summary

Panel	Treatment	Surface marker	Score fold change - shCD44 *vs.* shControl				
			Exp. 1	Exp. 2	Exp. 3	Average ± SD	p-value
Fig. 5-B1	No stimulation	Integrin β1	1.3	2.2	2.2	1.9 ± 0.5	0.10
Fig. 5-B2		CD24	1.3	1.5	2.2	1.7 ± 0.5	0.14
Fig. 5-B3	TME stimulation	Integrin β1	1.0	1.1	1.2	1.1 ± 0.1	0.19
Fig. 5-B4		CD24	0.5	0.5	1.6	0.9 ± 0.5	0.76

Supplementary Figure S6: CD44 knock-down reduces the proportions of the TME-enriched CD44+/β1+ and CD44+/CD24- sub-populations. (**A**, **B**) The panels demonstrate a representative experiment of *n* = 3, whose summary is presented in Figure 5A. In brief, following CD44 knock-down, TME Stimulation was performed and the proportions of CD44+/β1+ and of CD44+/CD24- cells were determined (Please see comment in the legend of Figure 5 on position of axes in CD24 staining). (A) Proportions of CD44+/β1+ cells. (B) Proportions of CD44+/CD24- cells. (**C**) The table summarizes the findings obtained in *n* = 3 experimental repeats, whose representative experiment is presented in Figure 5B. The data are expressed as a fold change in score (Mean fluorescence intensity X % Positive cells). Please note that in those cases in which there was a change in marker expression (β1 and CD24 expression upon CD44 down-regulation in non-stimulated cells), all repeats demonstrated the same trend but to differing extents. Therefore, despite the similar trend of the results, they were not statistically significant.

A shZeb1: CD44+/β1+ sub-population

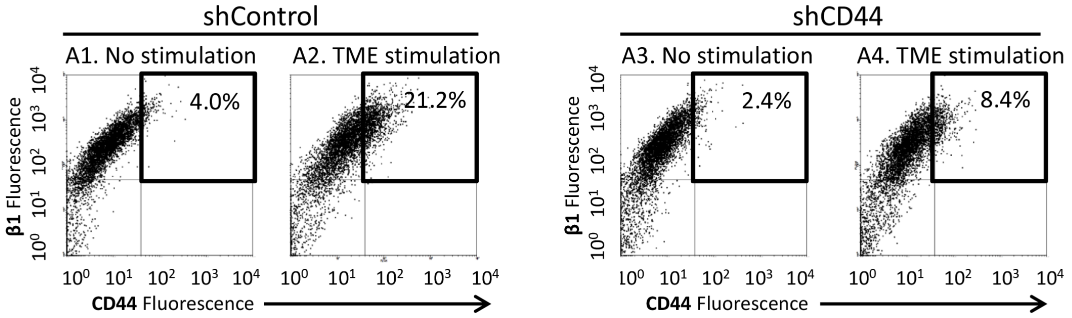

B shZeb1: CD44+/CD24$^{low/-}$ sub-population

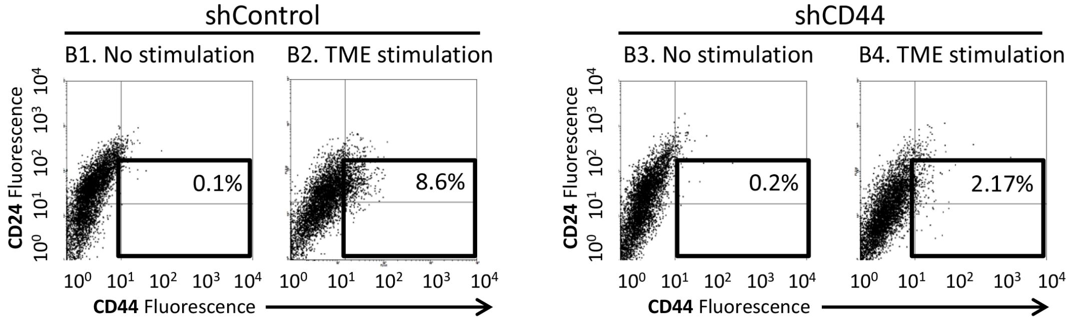

C shZeb1: CD44, β1 and CD24 expression - Summary

Panel	Treatment	Surface marker	Score fold change - shZeb1 *vs.* shControl					
			Exp. 1	Exp. 2	Exp. 3	Exp. 4	Average ± SD	p-value
Fig. 7-B1	No stimulation	CD44	1.7	1.4	0.2	0.5	0.9 ± 0.7	0.88
Fig. 7-B2		Integrin β1	0.9	0.8	1.0	1.1	0.9 ± 0.1	0.33
Fig. 7-B3		CD24	0.6	0.8	0.4	0.5	0.6 ± 0.2	0.07
Fig. 7-B4	TME stimulation	CD44	0.5	0.3	0.1	0.4	0.3 ± 0.2	0.02
Fig. 7-B5		Integrin β1	0.9	0.9	0.7	0.7	0.8 ± 0.1	0.04
Fig. 7-B6		CD24	0.5	0.7	0.5	0.5	0.6 ± 0.1	0.01

Supplementary Figure S7: Zeb1 knock-down reduces the proportions of the TME-enriched CD44+/β1+ and CD44+/CD24$^{low/-}$ sub-populations. (**A**, **B**) The panels demonstrate a representative experiment of n = 3, whose summary is presented in Figure 7A. In brief, following Zeb1 knock-down, TME Stimulation was performed and the proportions of CD44+/β1+ and of CD44+/CD24$^{low/-}$ cells were determined. (A) Proportions of CD44+/β1+ cells. (B) Proportions of CD44+/ CD24$^{low/-}$ cells. (**C**) The table summarizes the findings obtained in n = 4 experimental repeats, whose representative experiment is presented in Figure 7B. The data are expressed as a fold change in score (Mean fluorescence intensity X % Positive cells). Please note that in the case of CD24 expression in non-stimulated cells, all repeats demonstrated the same trend but to differing extents. Therefore, despite the similar trend of the results, they were not statistically significant.

Primary tumors: Titration

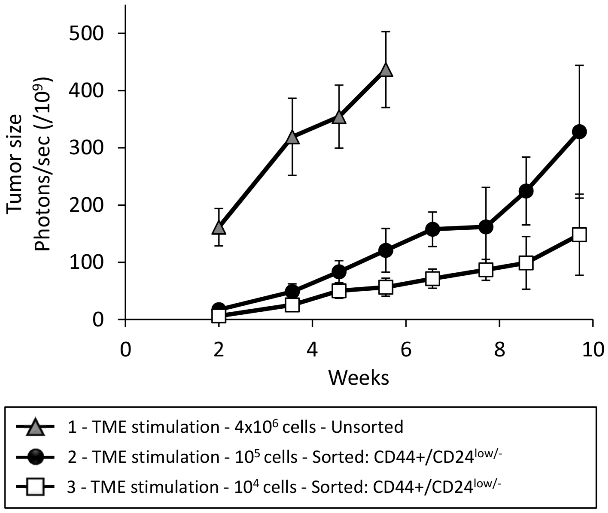

Supplementary Figure S8: Titration of inoculated MCF-7 cells in generating primary tumors in mice. mCherry-expressing MCF-7 cells were exposed to TME Stimulation (as in Figure 1, for three days in culture). Unsorted, TME-stimulated cells were inoculated to the mammary fat pad of female mice of Group 1, at concentration of 4×10^6 live cells/mouse. In parallel, following the same TME Stimulation, cells were sorted and CD44+/CD24$^{low/-}$ cells were inoculated to mice of Group 2 (10^5 live cells/mouse) and Group 3 (10^4 live cells/mouse). Primary tumors were followed in intact mice weekly for ~10 weeks by the IVIS™ intravital imaging system (whose signals, photons/sec, are divided by 10^9). The results are from a calibration experiment, performed on $n = 4$ mice/group.
